# ﻿Two new alkali-sink specialist species of *Paruroctonus* Werner 1934 (Scorpiones, Vaejovidae) from central California

**DOI:** 10.3897/zookeys.1117.76872

**Published:** 2022-08-15

**Authors:** Prakrit Jain, Harper Forbes, Lauren A. Esposito

**Affiliations:** 1 Institute for Biodiversity Science and Sustainability, California Academy of Sciences, 55 Music Concourse Drive, San Francisco, CA 94118 USA California Academy of Sciences San Francisco United States of America

**Keywords:** Conservation, desert, playa, salt flat, scorpions

## Abstract

Herein we describe two new species of *Paruroctonus* (Werner 1934) from California: *Paruroctonussoda***sp. nov.** from the Soda Lake playa at the center of the Carrizo Plain in San Luis Obispo county and *Paruroctonusconclusus***sp. nov.** from the Koehn Lake playa in the Mojave Desert of Kern County. They can be differentiated from other *Paruroctonus* by a combination of morphological features including deeply scalloped pedipalp fingers in males, specific patterns of fuscous pigmentation, unique setal counts, and unique morphometric ratios. They can also be separated from one another by the latter three characters. Photographs of a large selection of live scorpions are provided, including detailed images and figures of many morphological features. Their distributions, habitats, and ecologies are discussed; and important steps towards their conservation are described.

## ﻿Introduction

*Paruroctonus* Werner 1934 is the most speciose genus of scorpions in California. Including the two species described in this paper, it consists of 17 recorded species in the state. This includes wide-ranging generalists such as *Paruroctonussilvestrii* (Borelli, 1909), *P.becki* Gertsch & Allred, 1965, and *P.boreus* (Girard, 1854), as well as range-restricted specialists such as *Paruroctonusbantai* Gertsch & Soleglad, 1966, *P.arenicola* Haradon, 1984, and *P.shulovi* Williams, 1970. Most specialist desert species are restricted to habitats that retain more water than the surrounding environment. Examples found in California include sand dune specialists such as *Paruroctonusarenicolanudipes* Haradon, 1984, *P.xanthus* Gertsch & Soleglad, 1966, *P.hirsutipes* Haradon, 1984, and *P.baergi* Williams & Hadley, 1967 as well as playa/spring specialists such as *Paruroctonusbantai* Gertsch & Soleglad, 1966 (both ssp.).

The most recent large-scale taxonomic work on the genus *Paruroctonus* was conducted by Haradon in three publications in 1984 and 1985 (Haradon 1984a, b, 1985). He described several new species in *Paruroctonus*, including four from California (Haradon 1984a, b, 1985), and split the genus into three infragroups: *gracilior* group, *stahnkei* group, and *boreus* group. He then further split each of the latter two into an additional four microgroups (Haradon 1985). These groups have not been rigorously tested through empirical data, and morphological diagnoses for both infragroups and microgroups contain multiple exceptions (Haradon 1984a, b, 1985), making it impossible to accurately assign a new species to any of them. Phylogenetic evidence has also not yet supported any of these group distinctions, and in one case, has suggested that the microgroups in the *boreus* infragroup may be incorrectly constructed (Haradon 1985; Miller et al. 2014). Although some or many of these groups may be valid, we have not suggested an infragroup or microgroup placement for the two species described herein and advise against using these or other groups within *Paruroctonus* until more phylogenetic work is conducted on the genus.

**Figure 1.**
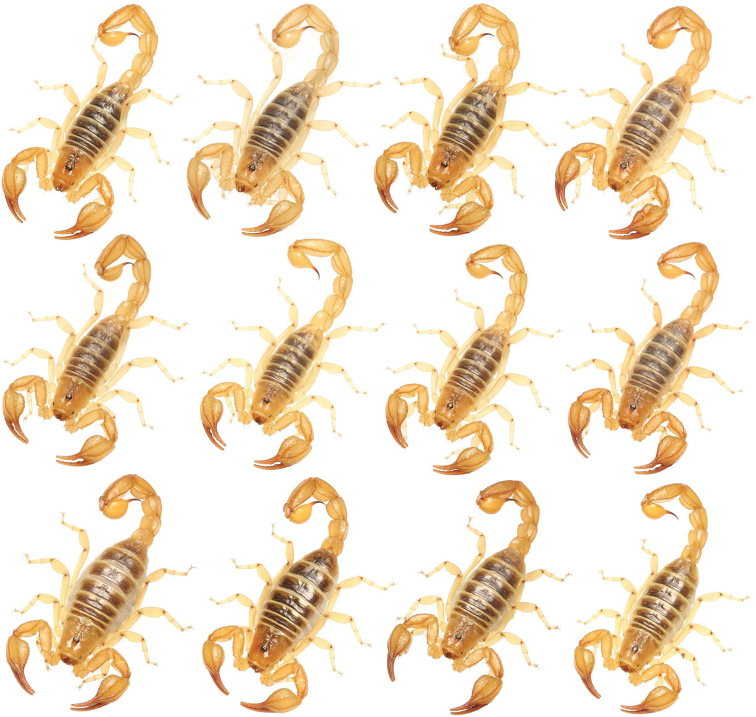
Variation of *Paruroctonussoda* sp. nov. from across their range. Top two rows, males; bottom row females; holotype male top left.

**Figure 2.**
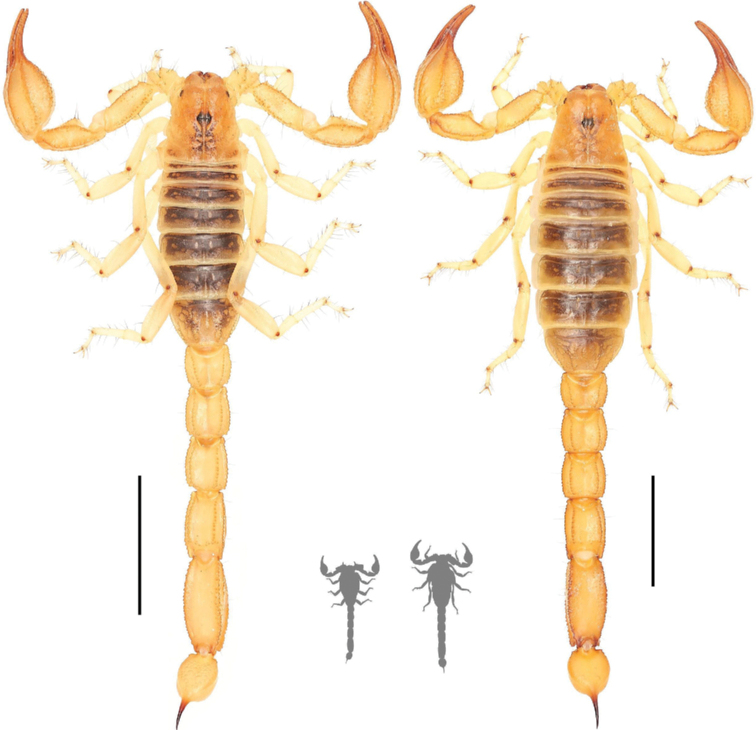
Dorsal habitus photographs of *Paruroctonussoda* sp. nov. holotype male (left) and female (right). Scale bars: 10 mm, silhouettes to scale.

**Figure 3.**
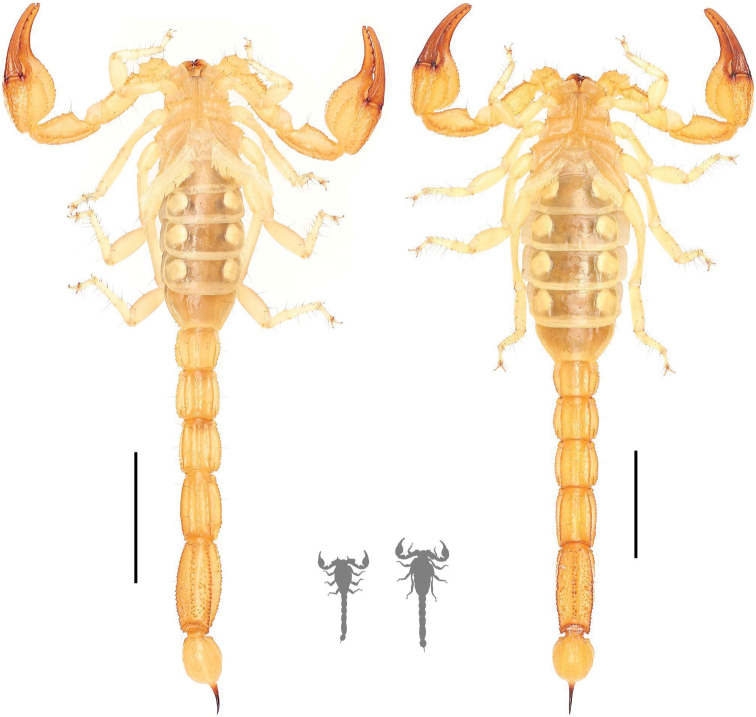
Ventral habitus photographs of *Paruroctonussoda* sp. nov. holotype male (left) and female (right). Scale bars: 10 mm, silhouettes to scale.

**Figure 4.**
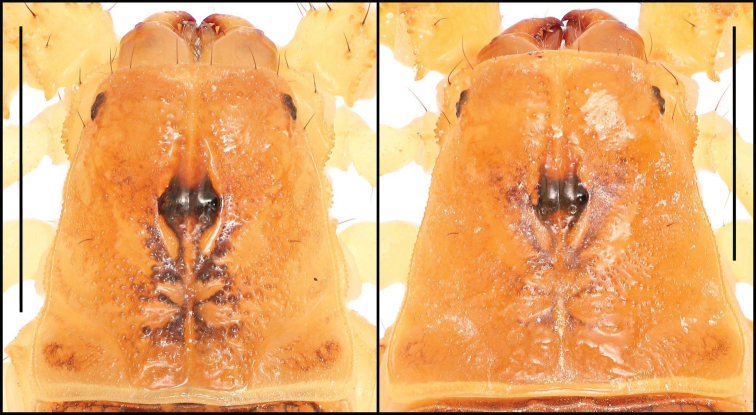
Carapace of *Paruroctonussoda* sp. nov. holotype male (left) and paratype female (right). Scale bars: 5 mm.

**Figure 5.**
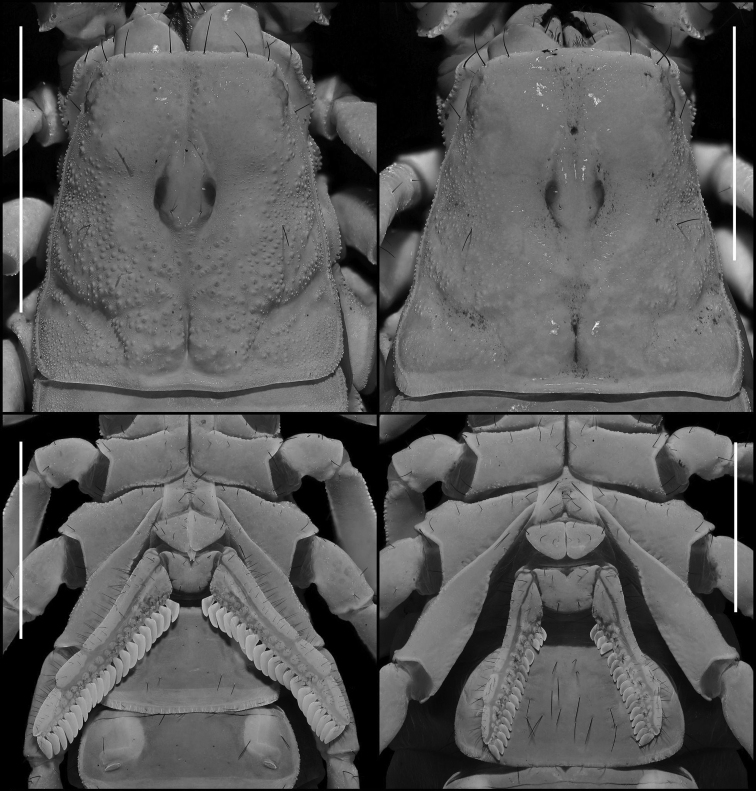
Carapace (upper) and sternopectinal region (lower) of *Paruroctonussoda* sp. nov. holotype male (left) and female (right). Taken under ultraviolet illumination. Scale bars: 5 mm.

**Figure 6.**
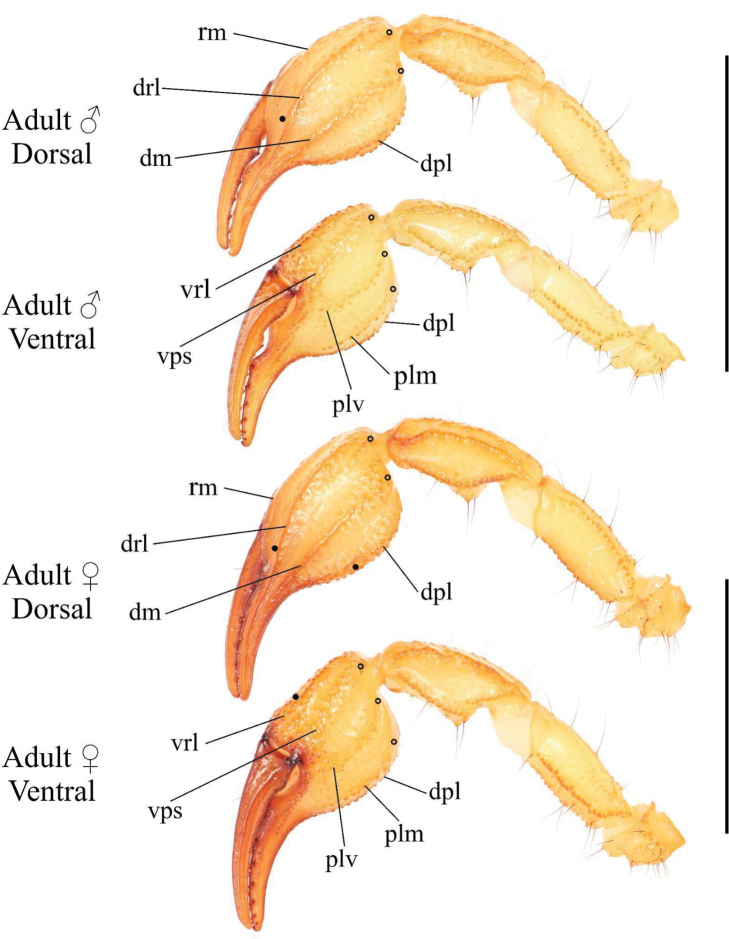
Pedipalp of *Paruroctonussoda* sp. nov., holotype male (above) and female (below). Macrosetae indicated with open circles (proximal) and closed circles (diagnosis character 4). Carinae abbreviations: retrolateral median (rm), dorsal retrolateral (drl), dorsal median (dm), dorsal prolateral (dpl), ventral retrolateral (vrl), ventral prosubmedian (vps), prolateral ventral (plv), prolateral median (plm). Scale bars: 10 mm.

**Figure 7.**
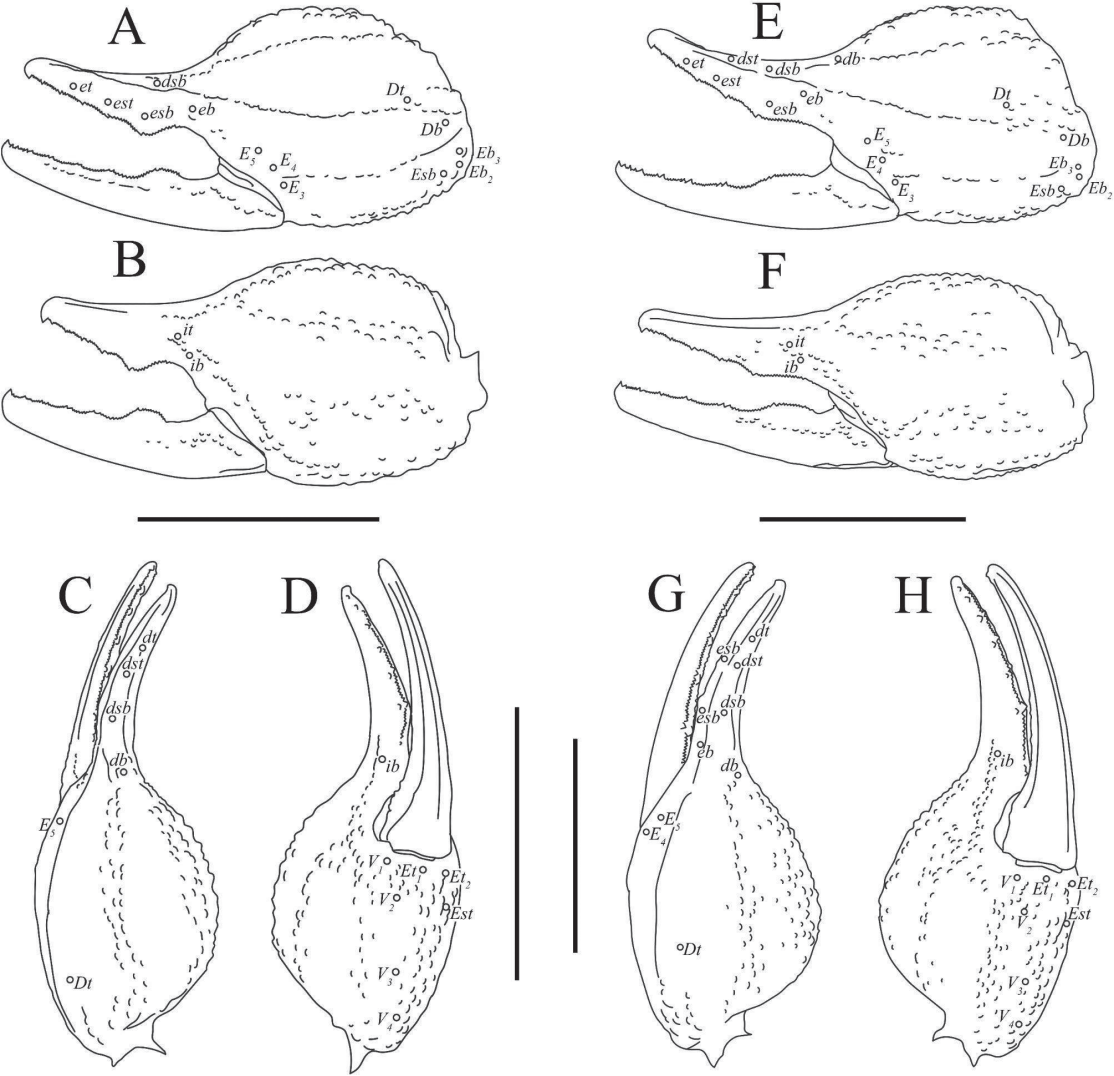
Illustrations of pedipalp chela of *Paruroctonussoda* sp. nov. **A–D** holotype male and **E–H** female **A, E** retrolateral **B, F** prolateral **C, G** dorsal **D, H** ventral. Trichobothria indicated with open circles. Scale bars: 5 mm.

**Figure 8.**
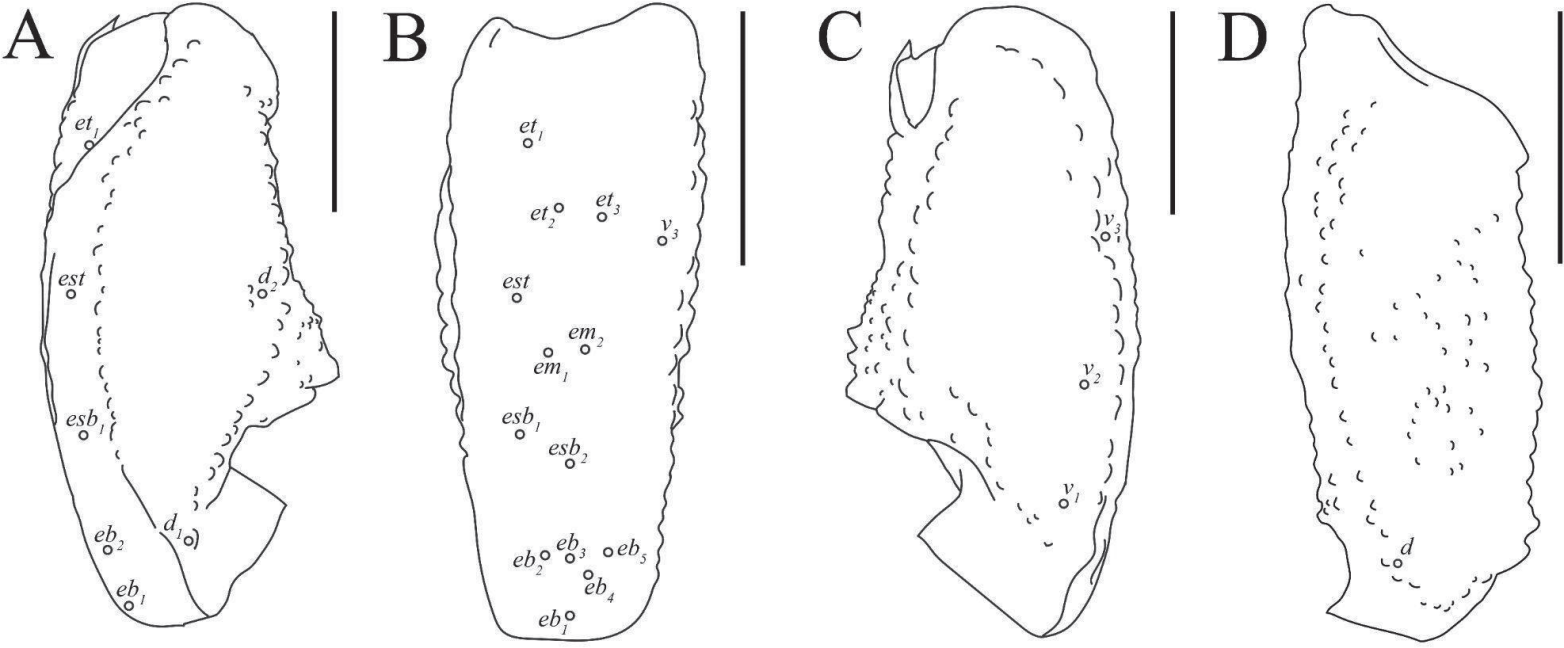
Illustrations of pedipalp patella and femur of Paruroctonussoda sp. nov. holotype male (**B, C**) paratype female (**A, D**). **A** dorsal patella **B** retrolateral patella **C** ventral patella **D** dorsal femur. Trichobothria indicated with open circles. Scale bars: 2 mm.

**Figure 9.**
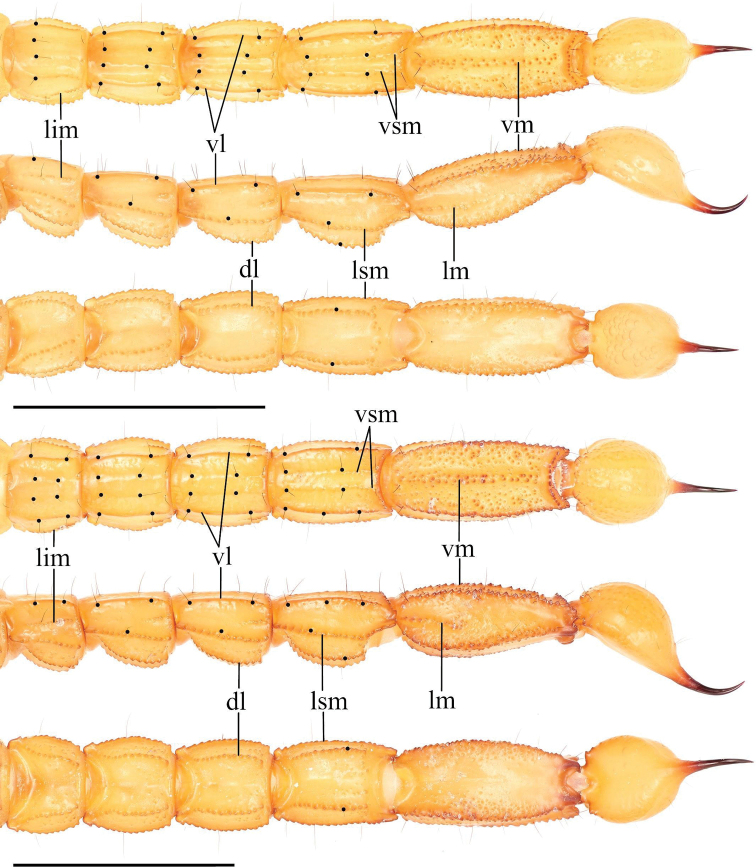
Metasoma of *Paruroctonussoda* sp. nov. holotype male (above) and female (below); ventral, lateral, and dorsal aspects (top to bottom). Ventral sub-median, ventrolateral, lateral submedian, and dorsolateral macrosetae on segments I-IV indicated with black circles (diagnosis character 3). Carinae abbreviations: Dorsolateral (dl), lateral median (lm), lateral supramedian (lsm), lateral inframedian (lim), ventrolateral (vm), ventral submedian (vs), and ventromedian (vm). Scale bars: 10 mm.

**Figure 10.**
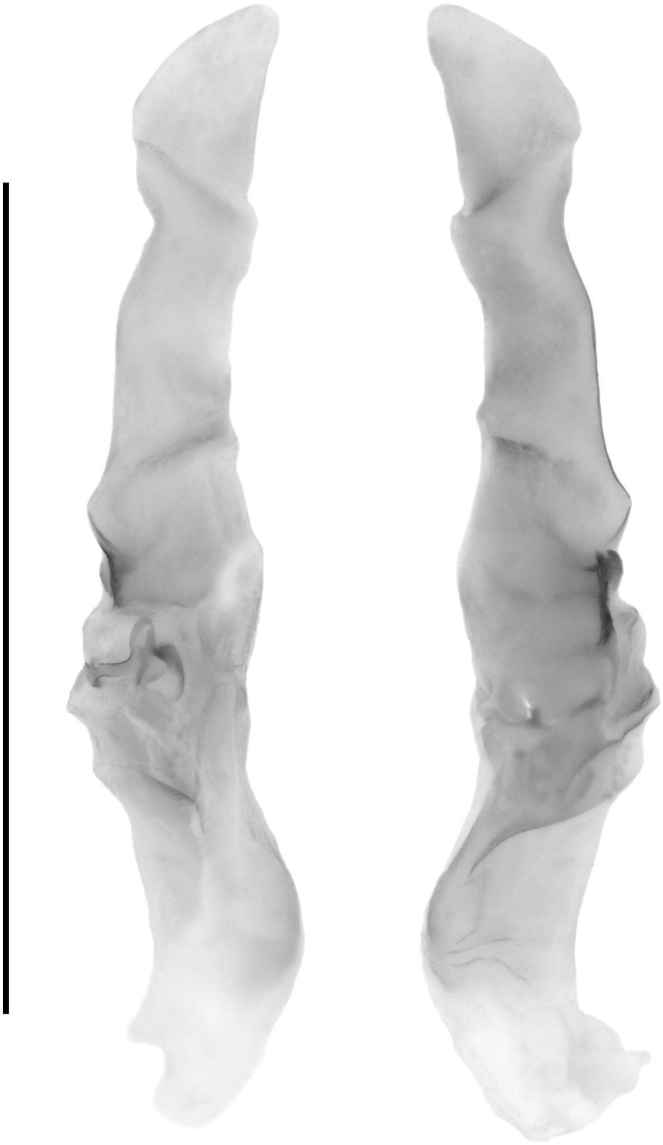
Right hemispermatophore of *Paruroctonussoda* sp. nov.: anterior aspect (left) and posterior aspect (right). Scale bar: 5 mm.

**Figure 11.**
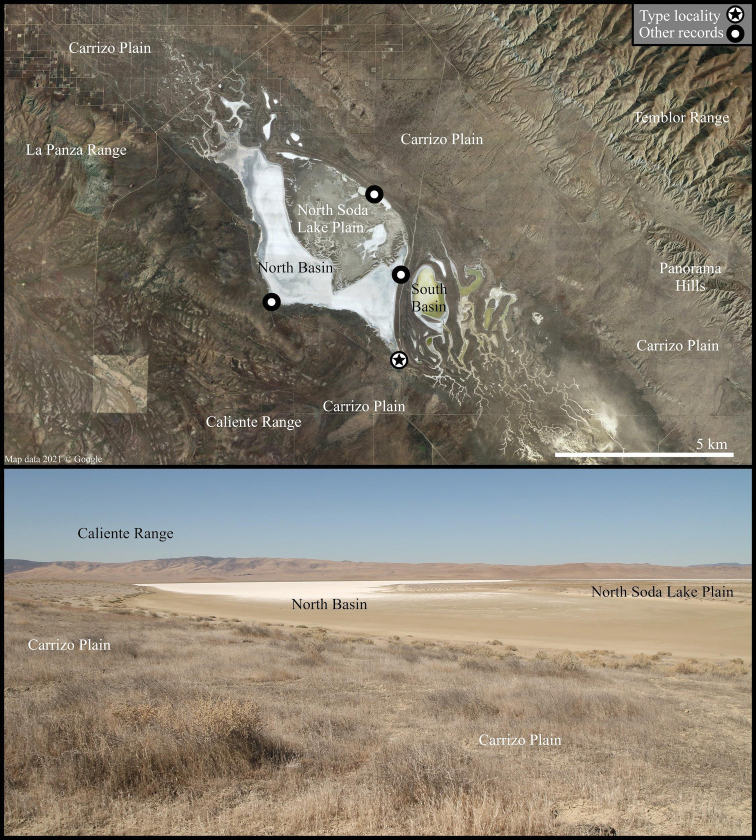
Soda Lake and the surrounding Carrizo Plain, the type locality of *Paruroctonussoda* sp. nov. Above, satellite imagery of the central Carrizo Plain taken in June 2019; below, a regional overview taken from the eastern side of the North Basin and facing southwest across the Soda Lake taken in May 2021.

**Figure 12.**
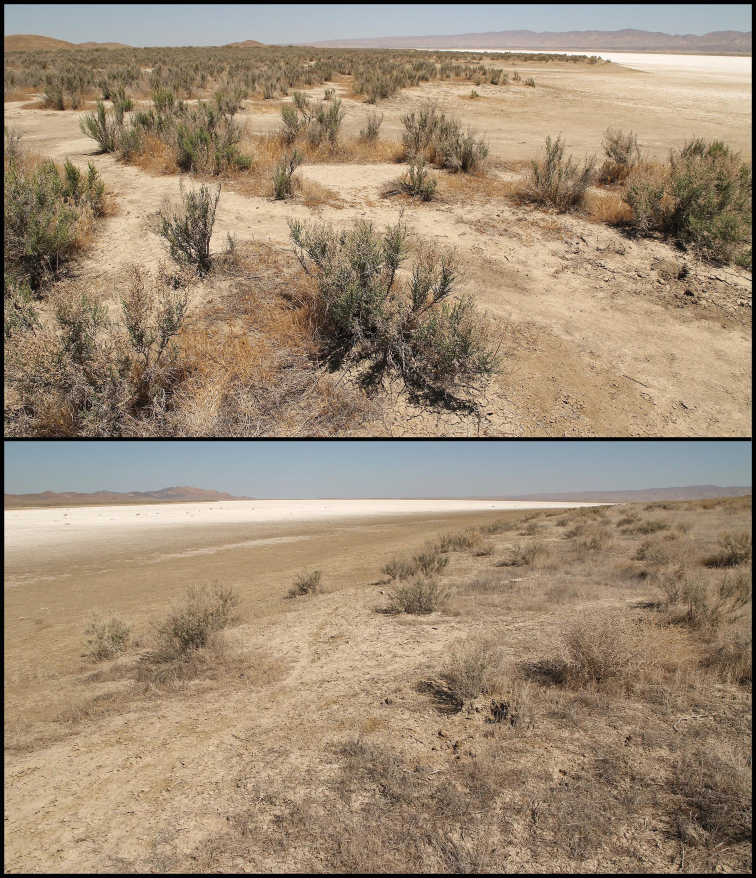
Habitat of *Paruroctonussoda* sp. nov. in May 2021. Note the dominance of *Allenrolfeaoccidentalis* and the soft, exposed clay soil along the lakebed.

**Figure 13.**
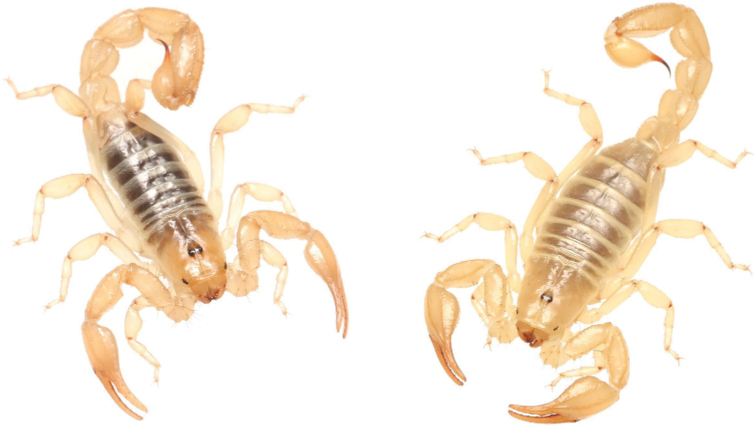
Photographs of a typical juvenile *Paruroctonussoda* sp. nov. (left), and atypical coloration observed in one late instar juvenile female. Not to scale.

**Figure 14.**
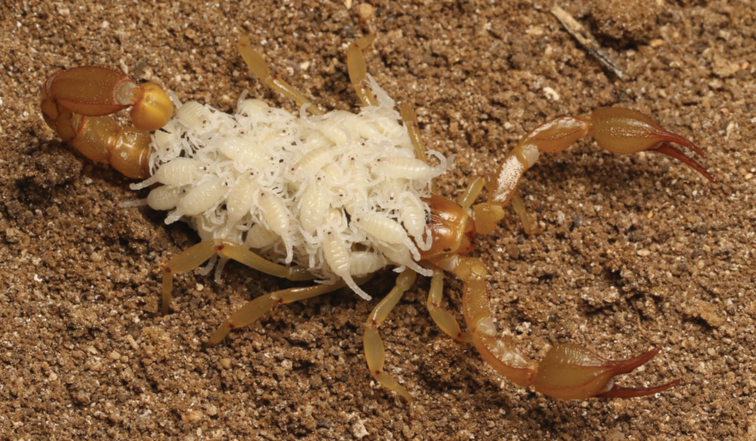
Adult female *Paruroctonussoda* sp. nov. with 51 newly born juveniles.

**Figure 15.**
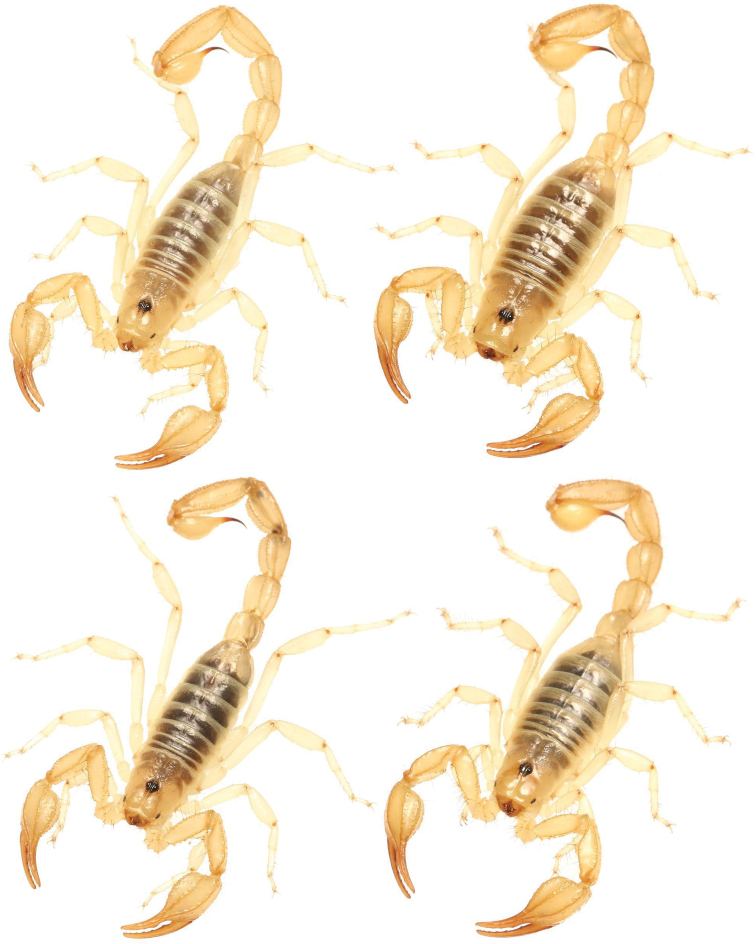
Photographs of four adult *Paruroctonusconclusus* sp. nov. illustrating intraspecific variation, holotype male is at top left.

**Figure 16.**
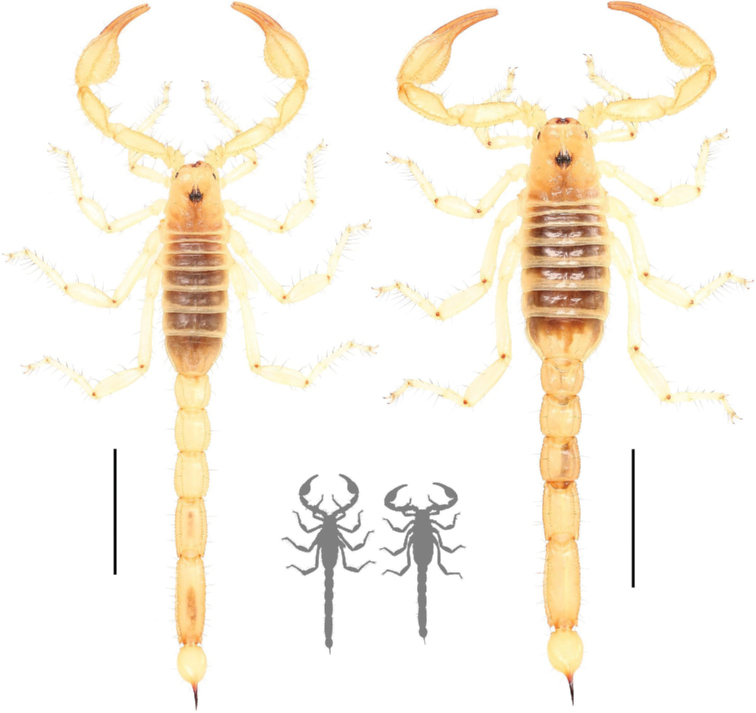
Dorsal habitus of *Paruroctonusconclusus* sp. nov. holotype male (left) and paratype female (right). Scale bars: 10 mm, silhouettes to scale.

**Figure 17.**
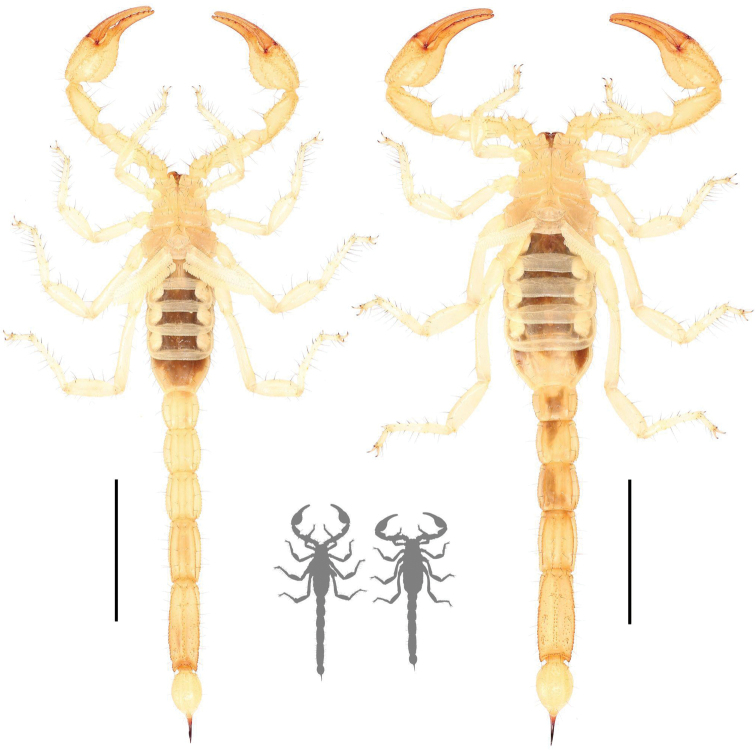
Ventral habitus of *Paruroctonusconclusus* sp. nov. holotype male (left) and paratype female (right). Scale bars: 10 mm, silhouettes to scale.

**Figure 18.**
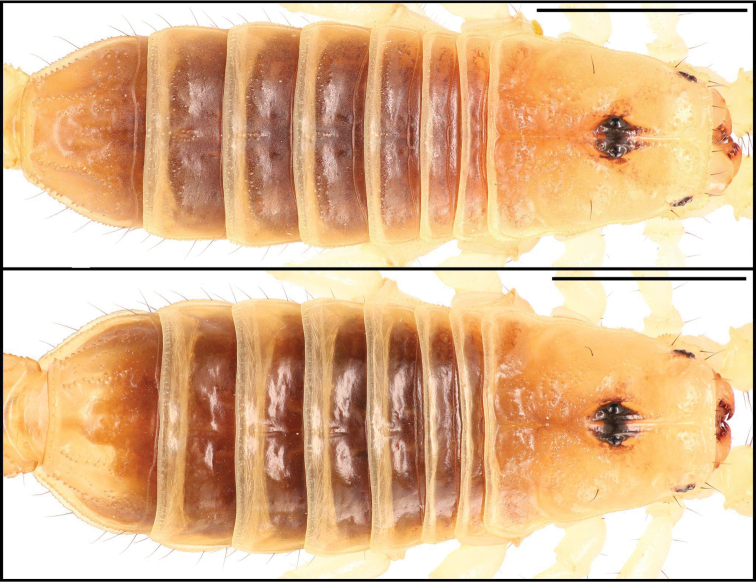
Dorsal trunk of *Paruroctonusconclusus* sp. nov. holotype male (above), paratype female (below). Scale bars: 5 mm.

**Figure 19.**
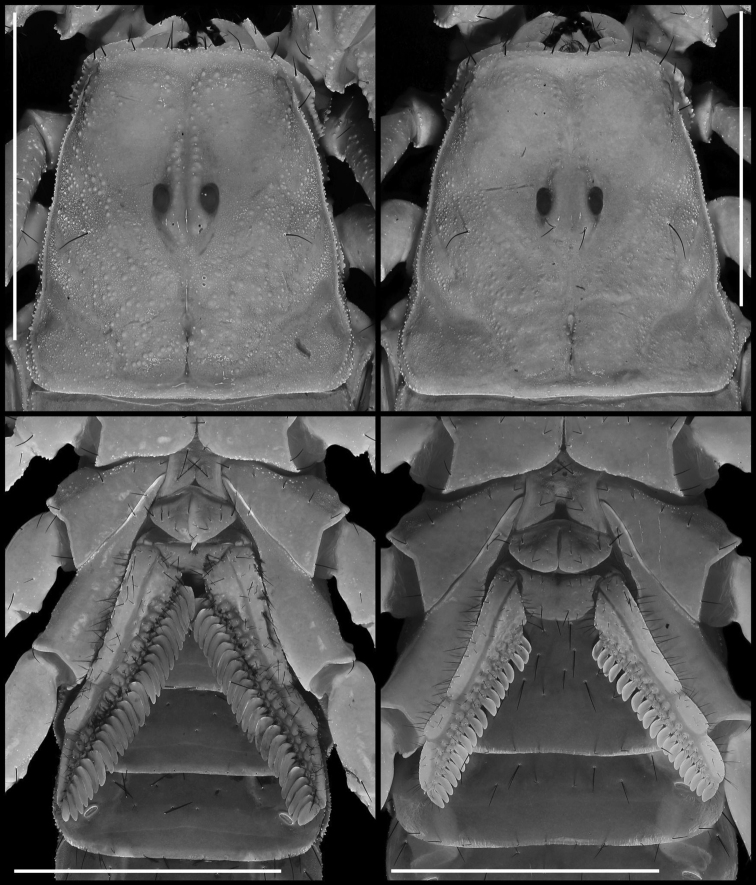
Carapace and sternopectinal region of *Paruroctonusconclusus* sp. nov. holotype male (left) and paratype female (right). Shown under ultraviolet illumination. Scale bars: 5 mm.

**Figure 20.**
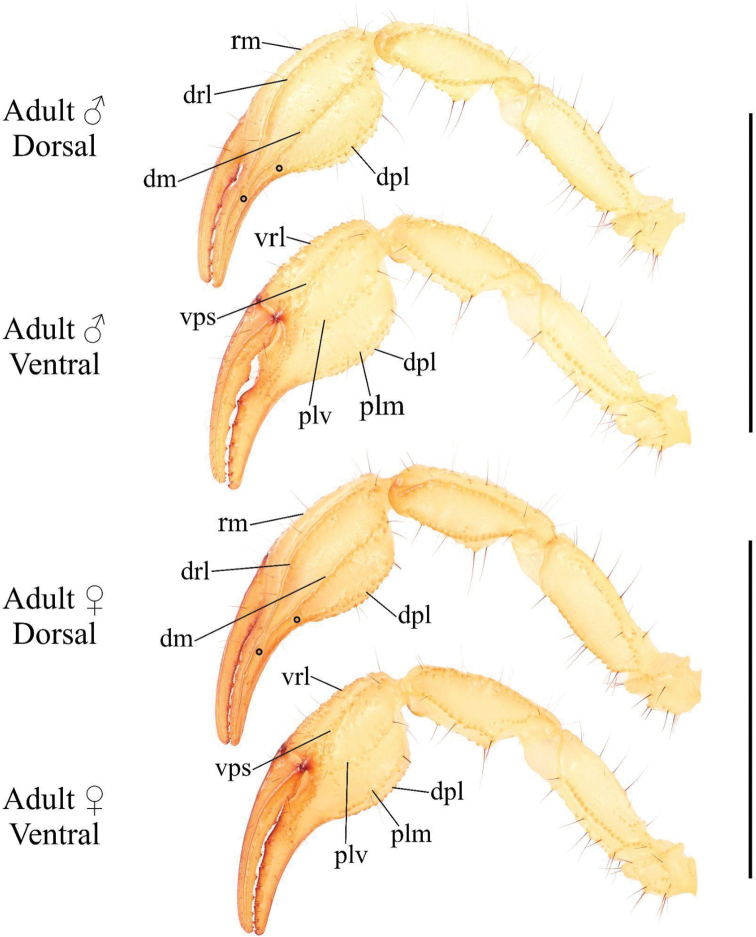
Pedipalp of *Paruroctonusconclusus* sp. nov., holotype male (above) and female (below). Trichobothria db and dsb (diagnosis character 8) indicated with closed circles. Carinae abbreviations: retrolateral median (rm), dorsal retrolateral (drl), dorsal median (dm), dorsal prolateral (dpl), ventral retrolateral (vrl), ventral prosubmedian (vps), prolateral ventral (plv), prolateral median (plm). Scale bars: 10 mm.

**Figure 21.**
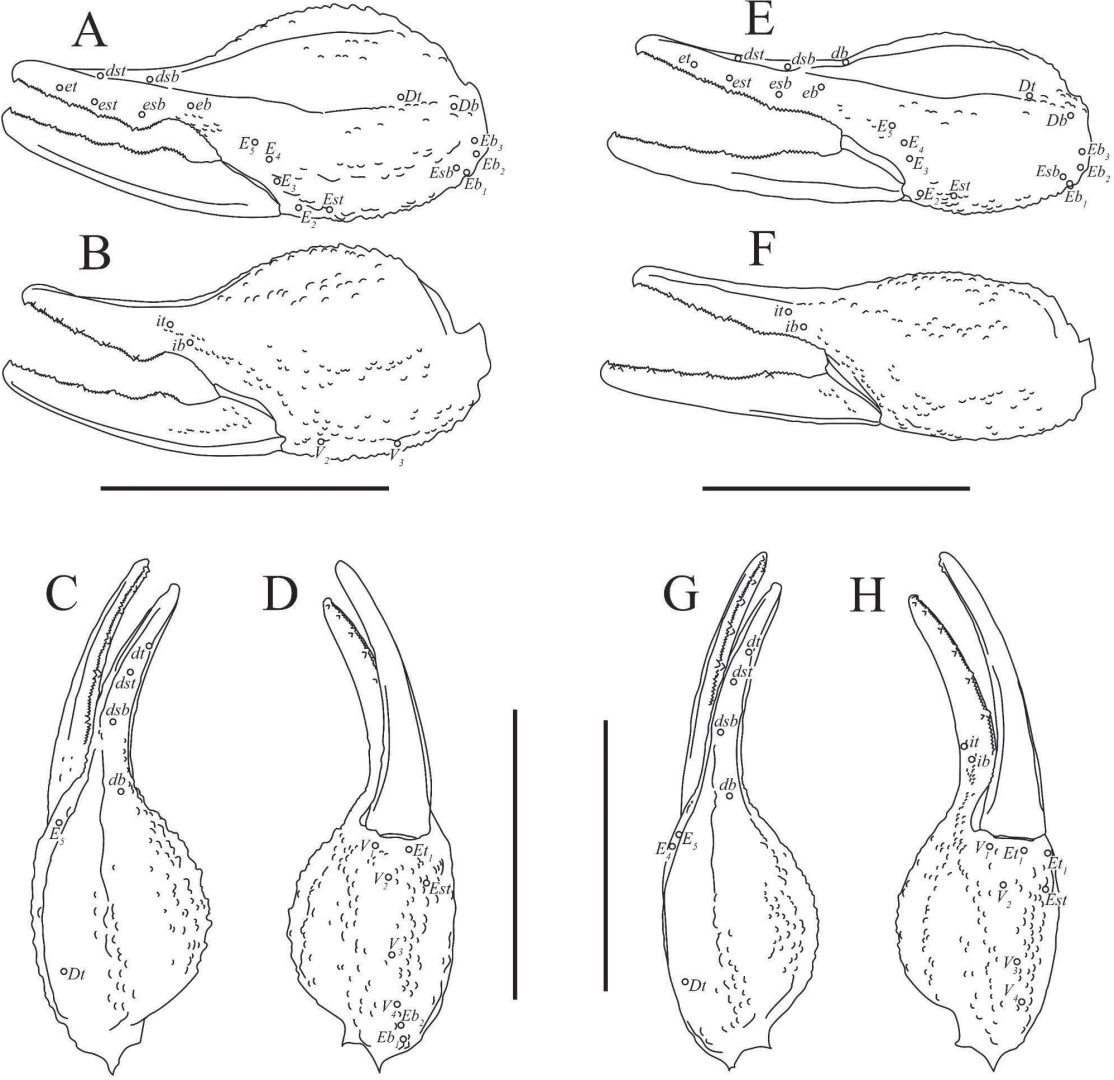
Illustrations of pedipalp chelae of *Paruroctonusconclusus* sp. nov. **A–D** holotype male **E–H** paratype female **A, E** retrolateral **B, F** prolateral **C, G** dorsal **D, H** ventral. Trichobothria indicated with open circles. Scale bars: 5 mm.

**Figure 22.**
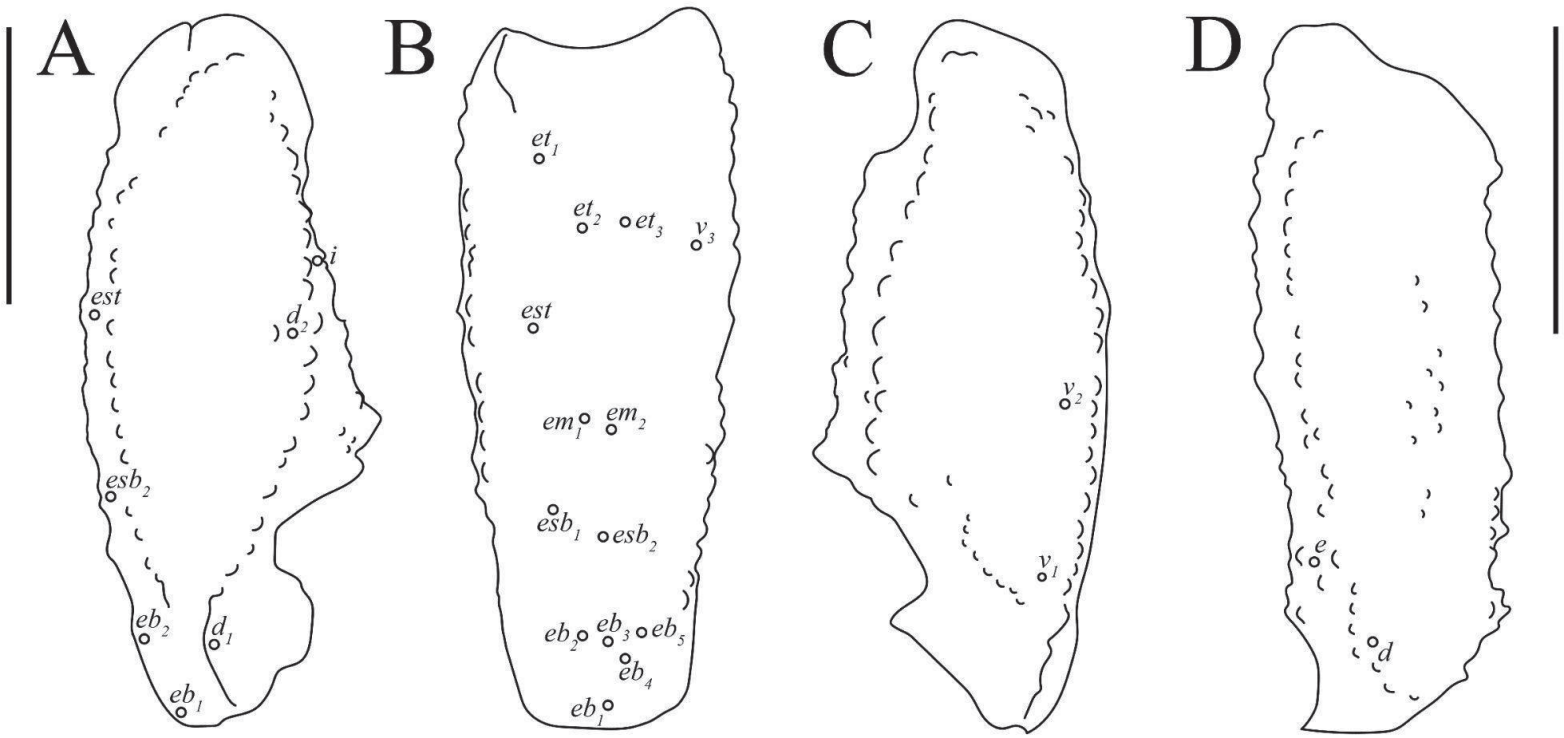
Illustrations of pedipalp patella and femur of *Paruroctonusconclusus* sp. nov. Holotype male, dorsal patella **A** retrolateral patella **B** ventral patella **C** dorsal femur **D** Trichobothria indicated with open circles. Scale bars: 2 mm.

**Figure 23.**
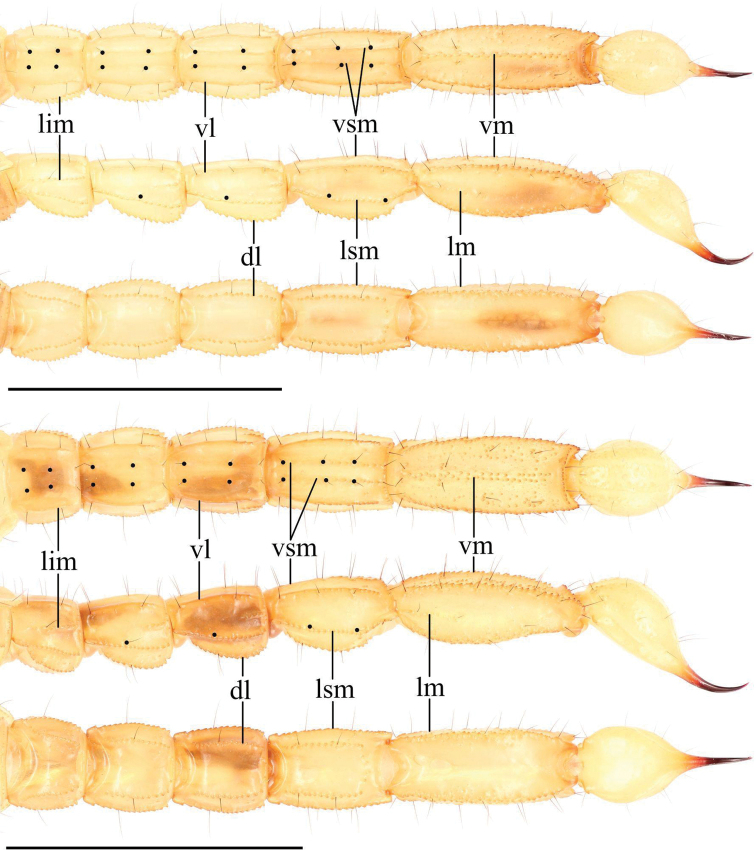
Metasoma of *Paruroctonusconclusus* sp. nov. male holotype (above) and female (below); ventral, lateral, and dorsal aspects (top to bottom). Ventral sub-median and lateral submedian macrosetae on segments I–IV indicated with black circles (diagnosis character 3). Carinae abbreviations: Dorsolateral (dl), lateral median (lm), lateral supramedian (lsm), lateral inframedian (lim), ventrolateral (vm), ventral submedian (vs), and ventromedian (vm). Scale bars: 10 mm.

**Figure 24.**
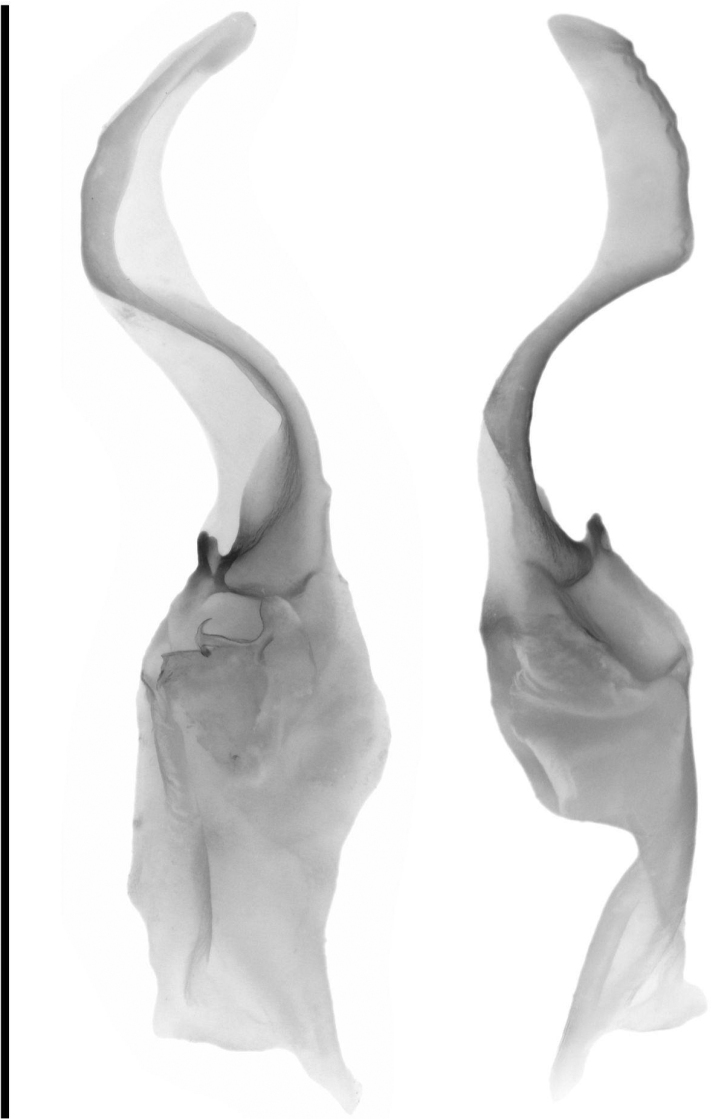
Right hemispermatophore of *Paruroctonusconclusus* sp. nov.: anterior aspect (left) and posterior aspect (right). Scale bar: 5 mm.

**Figure 25.**
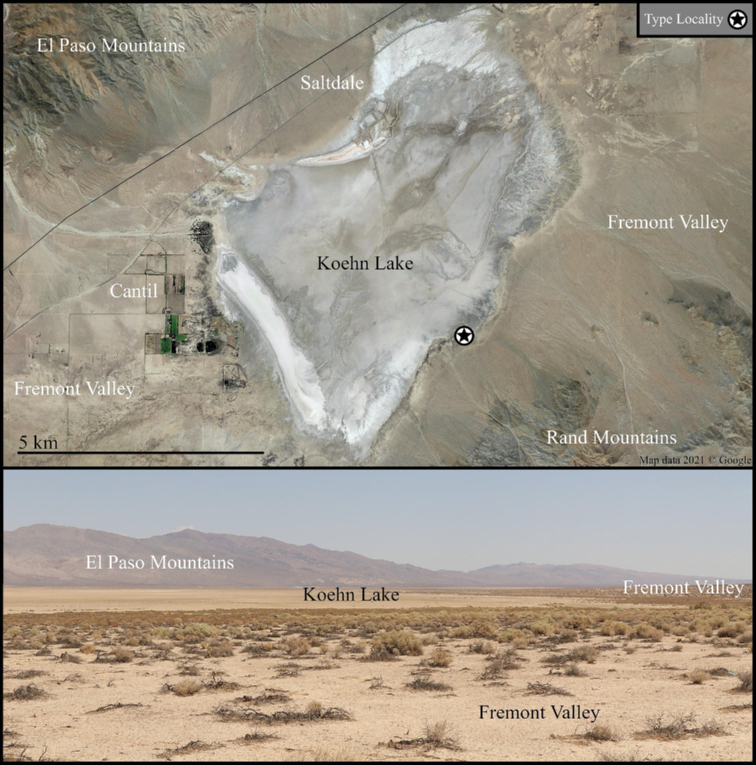
Koehn Lake and the surrounding Fremont Valley. Above, satellite imagery of Koehn Lake with the type locality of *Paruroctonusconclusus* sp. nov. indicated with a star, taken in September 2015. Below, a habitat overview looking north-northeast across Koehn Lake towards the El Paso mountains, taken in July 2021.

**Figure 26.**
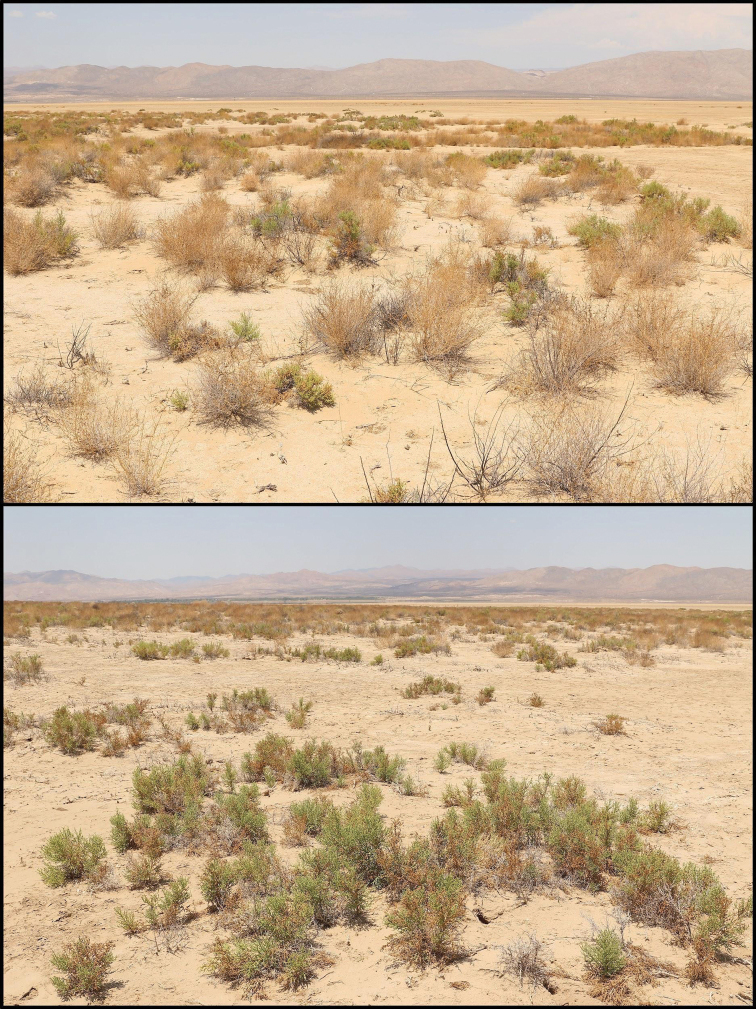
Habitat of *Paruroctonusconclusus* sp. nov. at the type locality, taken in July 2021.

**Figure 27.**
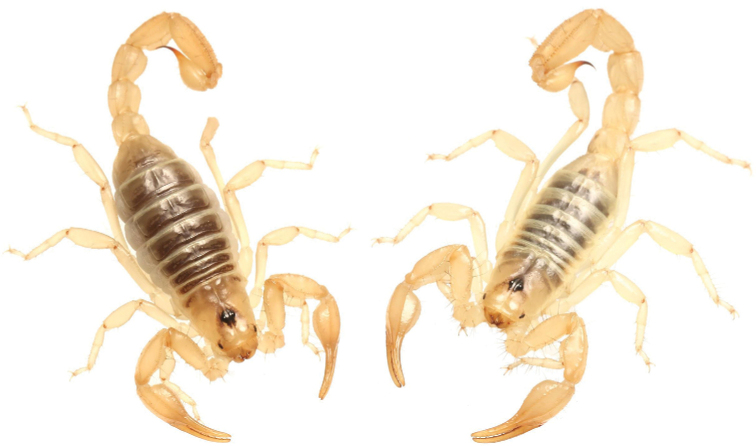
Early instar juvenile (left) and late instar juvenile (right) *Paruroctonusconclusus* sp. nov. Not to scale.

The two species described herein are specialist species restricted to alkali-sink environments surrounding desert playas: *Paruroctonussoda* sp. nov. (Figs 1–14) is found around Soda Lake in the Carrizo Plain, part of the San Joaquin Desert in San Luis Obispo county, and *Paruroctonusconclusus* sp. nov. (Figs 15–27) is found around Koehn Lake in the Fremont Valley, part of the Mojave desert in Kern county. Both the San Joaquin Desert and the Mojave Desert have an abundance of ephemeral water bodies, in large part due to several substantial rivers that flow into them from the surrounding mountains. We summarize the paleogeographic history of these playas and hypothesize what historic trends could predict for the future of these species. We also propose steps towards the conservation of *Paruroctonusconclusus* sp. nov., which should be considered threatened due to a restricted distribution, and discuss a possible instance of hypomelanism in *Paruroctonussoda* sp. nov. Lastly, we discuss the significance of morphological adaptations playa-specialist *Paruroctonus* have in common.

## ﻿Materials and methods

Specimens were photographed using a Canon EOS 7D camera with the Canon 100 mm F/2.8 macro lens. Habitat photos were taken using a Canon EOS 7D camera with the Canon 24–70 mm F/2.8 wide-angle lens or the Laowa 15 mm F/4 wide-angle macro lens. Stacked photographs were taken using the StackShot macro rail and were combined using Helicon Focus 7. Minor touch-ups to clean up the background and maintain even lighting were done using Gnu Image Manipulation Program and Adobe Photoshop. Satellite imagery for the maps is sourced from Google Earth and elevation data is sourced from NASA Shuttle Radar Topography Mission. Maps were constructed using QGIS, Gnu Image Manipulation Program, and Adobe Photoshop. Scale bars on figures are constructed using the pixel measurements in the photograph or traced illustration of the largest completely sclerotized precisely-measurable morphological feature parallel to the plane of the image and the corresponding length measurement on the scorpion.

Nomenclature and measurements largely follow Stahnke (1970) with a few exceptions. Basitarsal and telotarsal spine and setal nomenclature follows McWest (2009). Metasomal carinal nomenclature follows González-Santillán and Prendini (2013) with “lateral median” replaced with “lateral supramedian,” consistent with Sissom and Francke (1981) (Figs 9, 23). Metasomal setae follow the name of their associated carinae. Pedipalp and trichobothrial nomenclature follows González-Santillán and Prendini (2013) (Figs 6–8, 20–22). Setae on the manus follow the name of their associated carinae.

All elements in the diagnosis, unless otherwise noted, are not sexually dimorphic and apply to late instar juveniles as well. Counts and measurements separated by a “/” indicate a difference on the left/right sides of a single specimen, while those separated by a “–” indicate a range across multiple examined specimens. Setal counts used in the diagnosis are taken as the maximum number of macrosetae on either the right or left side of the individual scorpion (e.g., if a scorpion had two macrosetae on the left and three on the right, the setal count would be “3”). Total length includes telson and is not calculated additively. Measurements are made using digital calipers and are given in mm.

Specimens examined and photographed are either maintained alive in captivity or preserved in 95% ethanol. Preserved specimens examined are deposited at the California Academy of Sciences (**CAS**).

## ﻿Data resources

The data underpinning the analysis reported in this paper are deposited at GBIF, the Global Biodiversity Information Facility, and are available at https://doi.org/10.15468/zwgv36.

## ﻿Systematics

**Family Vaejovidae Thorell, 1876**

### ﻿Genus *Paruroctonus* Werner, 1934

#### ﻿ Paruroctonus soda sp. nov.

Taxon classificationAnimaliaScorpionesVaejovidae

E414E98A-8C39-5AED-BCD7-713F7178B8AC

https://zoobank.org/7BF88AF5-E85F-4627-ACE1-1ED7B1C5084C

Figs 1 , 2 , 3 , 4 , 5 , 6 , 7 , 8 , 9 , 10 , 11 , 12 , 13 , 14 , Table 1

##### Type material.

***Holotype***: USA • 1 ♂; California, San Luis Obispo County, southern tip of North Basin of Soda Lake; 35.2038, -119.8553; 585 m a.s.l.; 30 May 2021; collector leg Harper Forbes, Prakrit Jain; collected at night using handheld UV light; CASENT 9101932.

***Paratypes*.** USA • 1♂, 2♀; same data as for holotype; CASENT 9101933 • 1♂, 2♀; California, San Luis Obispo County, northeastern edge of North Soda Lake Plain; 35.2476, -119.8630; 587 m a.s.l.; 30 May 2021; collector leg Harper Forbes, Prakrit Jain; collected at night using handheld UV light; CASENT 9101934 • 1♂; California, San Luis Obispo County, western edge of North Basin of Soda Lake; 35.2186, -119.8958; 580 m a.s.l.; 30 May 2021; collector leg Harper Forbes, Prakrit Jain; collected at night using handheld UV light; CASENT 9101935.

##### Additional material examined.

USA • 1 ♀; California, San Luis Obispo County, eastern edge of North Basin of Soda Lake; 35.2263, -119.8548; 586 m a.s.l.; 30 May 2021; collector leg Harper Forbes, Prakrit Jain; collected at night using handheld UV light. • 2 ♂, 4♀; California, San Luis Obispo County, southern tip of North Basin of Soda Lake; 35.2038, -119.8553; 585 m a.s.l.; 30 May 2021; collector leg Harper Forbes, Prakrit Jain; collected at night using handheld UV light. • 1♂; California, San Luis Obispo County, northeastern edge of North Soda Lake Plain; 35.2476, -119.8630; 587 m a.s.l.; 30 May 2021; collector leg Harper Forbes, Prakrit Jain; collected at night using handheld UV light. • 7♂; California, San Luis Obispo County, western edge of North Basin of Soda Lake; 35.2186, -119.8958; 580 m a.s.l.; 30 May 2021; collector leg Harper Forbes, Prakrit Jain; collected at night using handheld UV light.

##### Diagnosis.

Differs significantly from other *Paruroctonus* species found in the San Joaquin Valley and its surrounding mountains (the Inner Southern Coast Range, the Sierra Nevada, the Tehachapis, and the northern mountains of the Transverse Range) by a combination of the following characteristics: 1: Fuscous markings entirely absent from the metasoma and the posterior margin of the tergites (Figs 1, 2). 2: Chelal fingers deeply scalloped in adult males, leaving a large proximal gap when closed. 3: Metasomal macrosetae along dorsolateral, ventrolateral, and ventral submedian carinae of segments I–IV follow the patterns 0,0,0,1; 1–2,2,2,2–3; and 1–2,2,2,2, respectively (Fig. 9). 4: All macrosetae on the manus greatly reduced in size; dorsal median, retrolateral median, ventral prosubmedian, prolateral ventral, and prolateral median carinae lacking any macrosetae except those at the proximal extent of their respective carinae (Fig. 6). 5: No large medial or distal retrolateral macrosetae on the pedipalp patella. 6: Length / Width ratios of metasomal segment V in adult males 2.22–2.59 and in adult females 2.21–2.28. 7: Chela length / Manus width and Chela length / Manus thickness ratios 2.05–2.16 and 2.96–3.14 in adult males, respectively and 2.15–2.30 and 2.98–3.14 in adult females, respectively.

##### Comparisons.

Comparisons are provided against other *Paruroctonus* sp. scorpions found in the San Joaquin Valley and its surrounding ranges, ordered ascendingly by the distance of the nearest record to the distribution of *P.soda* sp. nov. No *Paruroctonus* has been recorded within 13 kilometers of *P.soda* sp. nov., and while this distance could decrease significantly with more sampling, the habitat of *P.soda* sp. nov. is sufficiently distinct from that of any other nearby *Paruroctonus* that we consider sympatry to be unlikely.

*Paruroctonusvariabilis* Hjelle, 1982 differs from *P.soda* sp. nov. in the following characters relating to the numeration in the above diagnosis: (2) Chelal fingers not scalloped (straight), leaving a negligible proximal gap when closed. (3) Metasomal macrosetae along dorsolateral, ventrolateral and ventral submedian carinae on segments I–IV follow the patterns 0,1,1,2; 3,3–5,4–5,5–6; and 3–4,4,4–5,4–8, respectively. (4) Many large macrosetae on the manus; macrosetae along chelal dorsal median, retrolateral median, ventral prosubmedian, prolateral ventral, and prolateral median carinae, excluding any near the proximal extent of their respective carinae, follow the pattern 1–3,2–4,3–4,1–2,1–2. (5) Pedipalp patella with 3–5 large medial and 2 large distal retrolateral macrosetae. (6) length/width ratios of metasomal segment V in adult males 2.85–3.02, in adult females 2.63–2.89. (7) Chela length/manus width and chela length/manus thickness ratios in adult males 2.49–3.10, 3.39–4.03, respectively, in adult females 2.90–3.37, 3.94–4.27, respectively.

*Paruroctonussilvestrii* differs from *P.soda* sp. nov. in the following characters relating to the numeration in the above diagnosis: (1) Extensive fuscousity present on the ventral surface of the metasoma, mesosomal fuscousity extending to the posterior edge of the tergites. (2) Chelal fingers not scalloped (straight), leaving a negligible gap when closed. (3) Metasomal macrosetae along dorsolateral, ventrolateral, and ventral submedian carinae on segments I–IV follow the patterns 0,1,1,2; 2,3,3,3–4; and 2–3,3,3–4,3–4, respectively. (4) Many large macrosetae on the manus; macrosetae along chelal dorsomedian, retrolateral median, ventral prosubmedian, prolateral ventral, and prolateral median carinae, excluding any near the proximal extent of their respective carinae, follow the pattern 0–1,1–2,1–2,1,1. (5) Pedipalp patella with 2–4 large medial and 2 large distal retrolateral macrosetae. (6) Length/width ratios of metasomal segment V in adult males 2.72–2.90, in adult females 2.46–2.63. (7) Chela length/manus width and chela length/manus thickness ratios in adult males 2.59–2.70 and 3.36–3.65, respectively; in adult females 2.75–3.06 and 3.73–4.15, respectively.

*Paruroctonusboreus* differs from *P.soda* sp. nov. in the following characters relating to the numeration in the above diagnosis: (1) Fuscousity present on the ventral surface of the metasoma, especially on segments II–IV. (3) Metasomal macrosetae along dorsolateral, ventrolateral and ventral submedian carinae on segments I–IV follow the patterns 0,0–1,1,1–2; 2,3,3,3–4, and 2,2,2–3,3, respectively. (4) Several large macrosetae on the manus; macrosetae along chelal dorsal median, retrolateral median, ventral prosubmedian, prolateral ventral, and prolateral median carinae, excluding any near the proximal extent of their respective carinae, follow the pattern 0,1–2,1,1,1. (5) Pedipalp patella with 1–2 large medial and 2 large distal retrolateral macrosetae. (6) Length/width ratios of metasomal segment V in adult males 2.72–3.12, in adult females 2.50–2.71.

*Paruroctonusconclusus* sp. nov. differs from *P.soda* sp. nov. in the following characters relating to the numeration in the above diagnosis: (3) Metasomal macrosetae along dorsolateral and ventral submedian carinae on segments I–IV follow the patterns 0,1,1,2 and 2,2,2,3, respectively. (4) Several large macrosetae on the manus; macrosetae along chelal dorsal median, retrolateral median, ventral prosubmedian, prolateral ventral, and prolateral median carinae, excluding any near the proximal extent of their respective carinae, follow the pattern 0,1–2,1,1,1. (5) Pedipalp patella with 1 large medial and 2 large distal retrolateral macrosetae. (6) Length/width ratios of metasomal segment V in adult males 2.86–3.05, in adult females 2.47–2.56.

##### Description of male holotype.

***Coloration* (Figs 1–3).** Carapace orange-brown with faint fuscous markings present directly posterior to the median eyes, at the posterior-lateral corners of the carapace, and along the posterior edges of the interocular triangle. Tergites I–VI with fuscousity occupying the majority of the segment with the exception of the posterior and lateral margins; fuscousity somewhat reduced on VII. Legs pale cream to slightly tan. Pedipalps tan to orange with slightly darker orange carinae and dark orange fingers. Metasoma tan with faintly orangish carinae. Telson pale yellow, base of aculeus dark reddish, and aculeus black. Sternites brown, with tan spiracles. Pectines, sternum, and genital operculum tan to cream.

***Carapace* (Figs 4, 5).** Anterior margin roughly straight with three pairs of distinct macrosetae. Surface irregularly granular, with the largest granules near the center of the carapace. Very fine, evenly spaced granules present between the large granules. Lateral margins finely crenulate. Posterior median sulcus narrow and moderately deep, free of granulation. Anterior median, median ocular, lateral ocular, and posterior lateral sulci broad and shallow, entirely free of granules. Anterior and posterior marginal sulci shallow and sparsely granular. Median ocelli separated by a distance greater than the width of one ocellus. Three pairs of lateral ocelli present. Single pairs of macrosetae present posterior to the median ocelli, between the lateral ocelli and the margin of the carapace, and roughly halfway between the posterior median sulcus and the margin of the carapace, in line with the posterior edge of the ocular tubercle.

***Mesosoma* (Figs 2, 3).** Tergites I–VI very finely granular to smooth, except on the posterior and lateral thirds, which are weakly granular to granular. These areas become increasingly granular on subsequent segments. Median longitudinal carina absent on tergites I–II, indistinct and very weakly crenulate on III–VI. Submedian longitudinal sulci indistinct. One pair of small posterior sub-median setae on tergites I–VI. Tergite VII essentially smooth anteriorly and irregularly granular elsewhere. Posterior margin finely granular; lateral marginal, dorsolateral, and dorsal sub-median carinae crenulate. Median longitudinal carina indistinct. Sternites III–VI sparsely setose and smooth. Sternite VII smooth anteriorly, finely granular posteriorly, and granular laterally, with ventral submedian carinae indistinct and very weakly crenulate and lateral marginal carinae irregular and weakly crenulate.

***Genital operculum* (Fig. 5).** Sclerites roughly triangular with rounded corners, wider than long. Overlapping medially and separated slightly only at the posterior edge, with protruding genital papillae. Several macrosetae present on each sclerite.

***Sternum* (Fig. 5).** Type 2 with posterior emargination absent. Lateral lobes concave anteriorly, roughly straight laterally, convex posteriorly. Apex deep. Sclerite slightly wider than long, smooth to finely granular, especially along the slopes of the apex. Three pairs of macrosetae.

***Pectines* (Fig. 5).** Long, thin, and densely hirsute, with 21/21 tightly packed teeth on each side. Middle lamellae roughly circular distally, highly irregular in size and shape proximally; roughly 16/15 distinct and separated sclerotized sections are visible under ultraviolet illumination.

***Legs*. *Carinae*.** Retroventral carinae on Leg I femur unpigmented and finely crenulate; proventral carinae sparsely, finely and weakly crenulate on Leg I patella. Both decreasingly distinct on subsequent legs, proventral carinae on patella absent by leg IV. Other carinae indistinct to absent on all legs. On all legs, femur irregularly and very finely granular; other surfaces smooth.

***Telotarsi*.** Telotarsal retroinferior terminal macrosetae on legs I–IV 1/2, 2/2, 2/2, 2/2; other telotarsal retroinferior macrosetae on the distal half of telotarsi I–IV 1/1, 1/1, 1/1, 2/2. Two telotarsal retromedial macrosetae on each leg, with one always at the retromedial terminal position. Two large telotarsal retrosuperior macrosetae on each leg with consistent positions, with an additional smaller retrosuperior seta on dextral leg III. Single proinferior terminal macroseta on each leg except two on dextral leg II. Single proinferior distal macroseta on each leg, single other proinferior macroseta on legs II–IV except none on sinistral leg III. Two telotarsal promedial macrosetae on legs I–III at terminal and distal positions; one on leg IV in the terminal position. Two large telotarsal prosuperior macrosetae on each leg in terminal and medial positions. Single telotarsal superioterminal and superior macroseta present on all legs.

***Basitarsi*.** Three basitarsal spine rows present on legs I and II; proventral and retroventral spine rows equally dense and retrosuperior spine row less dense. The retroventral spine row extends ca. two-thirds the entire length of the segment, the proventral spine row extends through ca. half the segment, and the retrosuperior spine row extends through less than half. On leg III, proventral spine row absent and the retroventral and retrosuperior spine rows heavily reduced both in size and density. On leg IV, both the proventral and retroventral spine rows are absent and the retrosuperior spine row is heavily reduced in size and density, almost absent. Basitarsal retroventral macrosetae on legs I–IV, excluding only the distal retroventral spinoid macroseta at the end of the retroventral spine row, follow the pattern 2/3, 5/5, 4/5, 5/4, with variable sizes. Spinoid basitarsal proventral macrosetae on legs I–IV follow the pattern 2, 2, 3, 3; an additional thinner distal ventral macroseta is present on legs II–IV. Superior basitarsal macrosetae on legs I–IV consist of two spinoid macrosetae at the distal and mid retrosuperior positions; one macroseta at the distal prosuperior position; one macroseta at the distal superiomedian position adjacent to the distal retrosuperior macroseta, except on sinistral leg IV and dextral legs II–IV; and large superiomedian macrosetae following the pattern 4/4, 5/5, 5/5, 4/4. Prolateral macrosetae on legs I–IV, excluding one on the margin, follow the pattern 3/3, 3/3, 2/3, 2/2.

***Pedipalps* (Figs 6–8)**. ***Femur*.** Dorsal prolateral carina crenulate with two macrosetae on the proximal half; dorsal retrolateral carina also crenulate with two macrosetae on the proximal three-fourths. Dorsal surface sparsely granular. Retrolateral dorsosubmedian carina weakly crenulate; retrolateral surface otherwise smooth aside from a few proximal granules. Two long median macrosetae on the retrolateral surface. One large inframedian macroseta on the distal fourth of the retrolateral surface. Ventral retrosubmedian carina vestigial, irregularly granular with granules decreasing in size distally. Prolateral surface granular with two prolateral ventral macrosetae on the proximal half, one prolateral ventrosubmedian macroseta at the midpoint, and a pair of macrosetae on the distal margin.

***Patella*.** Dorsal retrolateral carina weakly crenulate with a large proximal macroseta; dorsal prolateral carinae crenulate with a small proximal macroseta. Dorsal surface essentially smooth. Retrolateral median carinae indistinct and very weakly crenulate, retrolateral surface otherwise smooth. Two very small and indistinct retrolateral distal marginal macrosetae present. Ventral retrolateral carina weakly crenulate; ventral prolateral and ventral median carinae crenulate. Ventral surface smooth. Prolateral median carina indistinct to absent, represented by a few large granules. Prolateral surface sparsely and weakly granular. Prolateral surface with large proximal supramedian, proximal inframedian, and distal inframedian macrosetae; heavily reduced distal supramedian macroseta. No large macrosetae present on the ventral and external surfaces.

***Chela*.** Dorsal prolateral carina indistinct, non-linear, and crenulate with no macrosetae, smooth on the fixed finger. Dorsal median carina weakly crenulate proximally and smooth distally, stopping at the base of the fixed finger, with a single small macroseta at its proximal extent. Dorsal retrosubmedian carina vestigial, consisting of only a few weak granules, and extending through less than the proximal fifth of the manus. Dorsal retrosubmedian accessory carina weakly crenulate, extending through less than the proximal fifth of the manus, with a small proximal macroseta. Dorsal retrolateral carina very weakly crenulate proximally and smooth distally, entirely smooth on the fixed finger, with a small distal macroseta near the base of the fixed finger. Retrolateral median carina very weakly granular and unpigmented, lacking setation. Ventral retrolateral carina indistinct and weakly crenulate, with 0/1 small macrosetae at its proximal extent. Intercarinal spaces on the dorsal and retrolateral surfaces smooth. Ventral prosubmedian carina indistinct and very weakly crenulate, with a single small macroseta at its proximal extent. Ventral surface smooth to granular near the base of the movable finger. Prolateral ventral and median carinae both crenulate to weakly crenulate with a single small macroseta at their respective proximal extents. Two additional small carinae are present near the base of the fixed finger, both of which are evenly and finely crenulate. Prolateral surface of the manus otherwise mostly smooth with some weak and irregular granulation in the distal half. The fingers are heavily scalloped, leaving a large proximal gap when closed. The chela is uniformly finely granular at the base of this gap. Retrolaterally, the fingers are smooth except some fine proximal granulation. Prolaterally, the fingers are smooth aside from a few patches of granulation on the proximal half. 19/16 small macrosetae and numerous microsetae are present on the ventral surface of the movable finger. No movable finger ventral prolateral, fixed finger prolateral median, or fixed finger prolateral dorsolateral macrosetae are present. The movable finger has one proximal prolateral median macroseta. A single proximal retrolateral median macroseta is present on the movable finger and a single dorsal prolateral seta is present near the distal end of the fixed finger. Both the fixed and movable fingers have five enlarged denticles dividing the primary denticles into six sub-rows, with an additional enlarged denticle at the distal extent of the movable finger, alongside the distal hook. On the fixed finger, rows I–VI contain 5/5, 7/6, 7/7, 7/8, 10/9, 12/10 primary denticles with a total row I–V count of 36/35. On the movable finger, rows I–VI contain 6/6, 8/8, 10/9, 9/10, 13/13, 9/10 primary denticles with a total row I–V count of 46/46. Each enlarged denticle as well as the distal finger-tip hook is accompanied by a single prolateral supernumerary denticle, for a total of six on the fixed finger and seven on the movable finger. There is a single macroseta posterior to each supernumerary denticle apart from the two most distal ones on each finger for a total of four on the fixed finger and five on the movable finger. Two further macrosetae are present near the proximal primary denticle row on the fixed finger.

***Metasoma* (Fig. 9).** Dorsal surface I–V smooth with a few scattered granules. Dorsolateral carinae on segments I–IV strongly crenulate to serrate, weakly crenulate on V. Lateral supramedian surface smooth with a few scattered granules. Lateral supramedian carinae I–IV crenulate. Lateral surface smooth. Lateral inframedian carinae crenulate on I–III, extend through only the posterior fifth of segments II–III. Lateral median carinae weakly crenulate on V, extending ca. a third of the way up the segment. Ventrolateral carinae I–IV smooth to weakly crenulate, becoming weakly crenulate on the posterior fourth of each segment. Ventrolateral carinae on segment V strongly crenulate to serrate. Ventral surface of segment I–IV smooth; ventral surface granular on segment V. Ventral sub-median carinae on I–IV smooth, unpigmented, and indistinct. Ventromedian carinae on segment V are crenulate, irregular, and disconnected. Dorsolateral macrosetae I–V follow the pattern 0,0,0,1,2. Lateral supramedian macrosetae I–IV follow the pattern 0,1,1,1. One Lateral median macroseta on V. Lateral inframedian macrosetae I–III follow the pattern 1,0,0. Ventrolateral macrosetae I–V, excluding any on the posterior margin of the segment, follow the pattern 1,2,2,2,3/4. Ventral submedian macrosetae I–IV, excluding those on the posterior margin of the segment, follow the pattern 1,2,2,2. Three pairs of macrosetae are present between the ventromedian and ventrolateral carinae on segment V. Two pairs of macrosetae on the ventral posterior margin of metasomal segment V; a single pair of macrosetae on the ventral posterior margins of other metasomal segment.

***Telson* (Fig. 9).** Very weakly granular on the ventral anterior portion, otherwise smooth. Sparsely setose ventrally and laterally.

***Hemispermatophore* (Fig. 10).** Hemispermatophore roughly equal in width from pedicel to stalk, three fold bauplan (Monod et al. 2017). Stalk wide and relatively straight and dorso-ventrally flattened. Distal carina and lamelar hook scletertized, lamelar hook bifurcate at terminus. Mating plug weakly scleretized, moderate in size with a wide bilobed base and relatively long stem terminating in a barb.

**Female.** Larger size. Relatively thinner chela with less curved fingers, weakly scalloped with a negligible gap when closed. Most proximal row on the chelal fixed finger with 16–21 primary denticles; most proximal row on the chelal movable finger with 10–13 primary denticles. Metasoma more robust. Pectines smaller overall with smaller teeth; teeth count 17–19 (17 n = 4.5, 18 n = 1.5, 19 n = 1) and middle lamella count 12–15 on a side. Sclerites separated narrowly through their entire length with the gap slowly increasing toward the posterior half.

**Variation. *Coloration* (Figs 1–3).** Fuscous markings posterior to the median eyes and in the posterior-lateral corners of the carapace range from typically prominent to sometimes indistinct to absent. Those along the edges of the interocular triangle are typically faint but range to absent. Fuscous markings on tergites I–VI also highly variable, ranging in extensiveness from covering the entire tergite except the posterior and lateral margins to covering only a small area around the anterior half of the submedian sulci. Fuscous markings on tergite VII also variable, ranging from covering approximately the anterior three-fourths of the segment to only being present in small areas along the dorsal submedian carinae. Other aspects of coloration in preserved specimens relatively consistent. In life, carapace, metasoma, and pedipalp coloration ranges from dark brown to tan, but is typically orange.

***Carapace* (Figs 4, 5).** Level and density of granulation variable. The elevated area of the interocular crescent on either side of the anterior median sulcus is sometimes largely free of granulation.

***Tergites* (Fig. 2).** Posterior granulation on tergites I–VI ranging from weakly granular through the posterior third of the segment to indistinct, very weakly granular, and restricted to the posterior margin of the segment. Lateral granulation sometimes absent.

***Pectines* (Fig. 5).** Pectines in males 21–24 (21 n = 2.5, 22 n = 5, 23 n = 5.5, 24 n = 2) with 15–20 middle lamellae per side.

***Legs*.** Retroventral carinae on the leg patella ranging from finely crenulate to very weakly crenulate, almost absent. Prosuperior carinae on the leg femur ranging from very finely crenulate to weakly finely crenulate, almost absent. Retroventral spine row on basitarsus III ranging from equal in length and density to retrosuperior spine row to indistinct, almost absent. Terminal retroinferior macrosetae on telotarsus II 1–2, other retroinferior macrosetae on telotarsus III 1–2, retromedian macrosetae on telotarsus IV 2–3. Additional small retrosuperior macrosetae present occasionally on legs II–III. Other large telotarsal retrolateral macrosetae described in the holotype description consistent with the exception of occasional asymmetrical additions or deletions. Second promedian macroseta occasionally present on leg IV and third promedian macroseta occasionally present on leg I; other large telotarsal prolateral macrosetae described in the holotype description consistent with the exception of occasional asymmetrical additions or deletions. Number of retroventral basitarsal setae on legs I–IV highly variable, within the following ranges for legs I–IV: 3–4, 5–6, 5–6, 5–6 with occasional asymmetrically added or missing setae. Proventral basitarsal macrosetae consistent. Large superior basitarsal macrosetae on legs I–IV, excluding the large spinoid distal and mid retrosuperior macrosetae; the large distal prosuperior and sometimes present small medial prolateral macrosetae; and the often absent macroseta at the distal superiomedian position adjacent to the distal retrosuperior macroseta, are highly variable, within the ranges 4–5, 5–6, 5–6, 4–5 with occasional asymmetrical deletions or additions of small macrosetae. Prolateral macrosetae on legs I–IV, excluding one on the margin, highly variable and often non-linear, within the ranges 3, 2–4, 2–4, 2–4 with occasional asymmetrical deletions. The smaller distal superiomedian macroseta is often missing on any leg.

***Pedipalps* (Figs 6–8).** Macrosetae on femur variable: prolateral ventrosubmedian sometimes missing; retrolateral dorsosubmedian excluding those on distal margin 2–4; other occasional asymmetrical deletions. Proximal macroseta on the pedipalp patella dorsal prolateral carina small or large, other large macrosetae on patella consistent. Patella retrolateral median carina weakly crenulate to very weakly crenulate with an inconsistent pattern. Two very small and indistinct distal median macrosetae sometimes present on the external surface of the pedipalp patella; no large medial or distal macrosetae ever present on the external surface of the pedipalp patella. On the chela, a small median macroseta on the dorsal prolateral carina and the ventral retrolateral carina sometimes present. A small proximal macroseta on the ventral retrolateral carina and a small macroseta along the dorsal retrolateral carina near the base of the fixed finger sometimes absent. On the fixed finger, prolateral median macroseta rarely present. Ventral macrosetae on the movable finger 15–19. Number of primary denticles in rows I–V on the fixed finger within the ranges 3–6, 5–7, 6–8, 7–9, 9–12. Number of primary denticle in row VI on the fixed finger of males 9–14. Number of primary denticles in rows I–VI on the movable finger within the ranges 4–7, 6–9, 8–10, 10–12, 11–17. Number of primary denticle in row VI on the movable finger of males 8–10. Primary denticles on the fixed finger excluding those on the proximal row 30–38 and primary denticles on movable finger excluding those on the proximal row 42–50 with no obvious sexual dimorphism in either.

***Metasoma* (Fig. 9).** Crenulation on metasomal ventrolateral carinae variable, ranging from very weakly crenulate on the posterior third to weakly crenulate on the posterior half. Lateral inframedian carina on II–III from ca. one fifth to one third the length of the segment. Dorsolateral medial macroseta on V ranging from indistinct to absent to small but distinct for a total of 2–3 macrosetae. Ventrolateral macrosetae on I 1–2, ventral submedian macrosetae on I 1–2, ventrolateral macrosetae on IV 2–3, ventrolateral macrosetae on V 3–6, and lateral supramedian macrosetae on IV 1–2. Other metasomal setae are consistent with the exception of occasional asymmetrical deletions.

##### Remarks.

The most valuable taxonomic characters for *P.soda* sp. nov. are:

1. The macrosetal patterns on the pedipalps and metasoma are very consistent and unique, provide excellent diagnostics against almost all other *Paruroctonus*.
2. The morphometric ratios of different aspects of the metasomal segments and chela are fairly consistent and do not overlap with those of several other *Paruroctonus*.
3. The lack of fuscous markings on the metasoma and chelae is very consistent and provides a helpful diagnostic for comparison with several other *Paruroctonus*.
4. The overall color pattern and the fuscous patterning on the carapace and tergites is somewhat variable but is still a reliable diagnostic character.

Other taxonomic characters which may be taxonomically valuable in some cases, but are typically not useful, include:

1. The telotarsal macrosetae are somewhat variable but have different counts than those of certain other *Paruroctonus*.
2. The extent of granulation on the carapace and tergites is fairly variable but is notably different from certain other *Paruroctonus*. This character, however, can be difficult to quantify.
3. The basitarsal macrosetae are generally extremely variable and are only helpful for differentiating *P.soda* sp. nov. from psammophilous *Paruroctonus*. The basitarsal spinoid distal and mid retrosuperior macrosetae are not variable but are still only helpful for differentiating *P.soda* sp. nov. from these psammophiles.
4. The granulation on the pedipalps, legs, and metasoma is somewhat variable and difficult to quantify. It is fairly similar to that of most other *Paruroctonus* species, although in isolated examples may be used for diagnosis.
5. The pectinal tooth counts are somewhat variable and are only useful as a diagnostic against some other *Paruroctonus*. Middle lamellae counts are also not taxonomically valuable, as they are typically ambiguous.
6. The chelal primary denticle counts are somewhat variable and overlap with those of most other *Paruroctonus*.

##### Habitat, distribution, and ecological notes.

*Paruroctonussoda* sp. nov. is known only from the area immediately surrounding Soda Lake in the Carrizo Plain, an area of the San Joaquin Valley in San Luis Obispo county, California (Fig. 11). Soda Lake is a complex of a single large and dozens of smaller typically dry lake beds draining the Carrizo Plain and its surrounding ranges, an endorheic watershed ca. 1230 km^2^ in size (Stephenson 2013). The lake complex began to form during the Pliocene epoch when tectonic activity severed a connection to the ocean (Cooper 1990; Stephenson 2013). A permanent, deep, brackish lake persisted from ca. 75 kya to around 16–17 kya, at which point a drying climate and hotter temperatures led to the lake shrinking and increasing in alkalinity (Stephenson 2013). The habitat surrounding the lake is now an alkali sink largely dominated by *Atriplexspinifera*, *Atriplexvallicola*, *Allenrolfeaoccidentalis*, and various small wildflower and grass species (Munz and Keck 1949; [Bibr B1][Bibr B23]).

The Carrizo Plain receives approximately 230 mm of sporadic winter rain in an average year resulting in an arid climate. Water drainage from the surrounding Temblor, La Panza, and Caliente ranges, which receive a slightly greater amount of rainfall, keeps the Soda Lake complex and the immediately surrounding area comparatively moist (Stephenson 2013). The summer climate in the region is hot and arid, with little to no rainfall and temperatures typically in excess of 35 °C during the hottest months.

Along the western, southwestern, and eastern edges of the largest basin (North Basin), we found *Paruroctonussoda* sp. nov. to be present only in a thin band of soft clay soil dominated by *Allenrolfeaoccidentalis* immediately adjacent to the edge of the dry lakebed (Figs 11, 12). To the northeast of the North Basin is a relatively large area of soft clay soils including a multitude of smaller basins (North Soda Lake Plain). Specimens of *Paruroctonussoda* sp. nov. were collected at the point in the North Soda Lake Plain region furthest from the edge of the North Basin, suggesting that it is likely found throughout a significant portion of the North Soda Lake Plain. *Paruroctonussoda* sp. nov. was not found along the edge of the second-largest basin (South Basin) and the smaller basin immediately to its north despite significant sampling. We hypothesize that *Paruroctonussoda* sp. nov. is absent from these basins because the band of soft clay soil surrounding these basins is too narrow to support a population of this species. It is, however, impossible to make high-confidence conclusions of absence from a single night of sampling. *Paruroctonussoda* sp. nov. was also not found in any of the areas of relatively tougher soil dominated by *Atriplexpolycarpa* or *A.spinifera*. We hypothesize that this species’ reliance on soft clay soils may be due to the high summer surface temperatures in the area, as the softer soils form deep cracks and are relatively easier to burrow into.

No scorpions were found in sympatry with *Paruroctonussoda* sp. nov. However, the presence of *Hadrurusobscurus* Williams, 1970 and *Paravaejovis* sp. is possible, as both species have records from the Panorama Hills a few kilometers from Soda Lake and the latter has records a short distance to the north of the North Basin. The geographically closest *Paruroctonus* is *P.variabilis*, found in the nearby Panorama Hills and Temblor range. However, we consider it unlikely that *P.variabilis* is found in the flat portion of the Carrizo Plain near Soda Lake as we have been unable to locate any after significant sampling.

**Table 1.** Table of measurements of 4 adult male and 4 adult female *Paruroctonussoda* sp. nov., in mm.

	Holotype male	Paratype male	Paratype male	Paratype male	Paratype female	Paratype female	Paratype female	Paratype female
CASENT#	9101932	9101934	9101933	9101935	9101934	9101934	9101933	9101933
Total L	49.54	45.23	50.20	41.95	60.99	61.28	59.71	56.08
Carapace L	5.95	5.89	6.59	5.33	7.47	7.27	7.41	7.55
Prosoma posterior W	5.41	5.89	6.16	5.15	7.16	7.04	7.28	8.18
Prosoma median W	4.40	4.33	5.02	4.29	5.92	5.77	5.65	5.77
Mesosoma L	13.12	10.62	12.80	11.85	18.47	21.75	18.89	13.21
Metasoma L	30.10	28.29	30.65	25.80	33.67	33.01	34.25	34.32
Metasoma I L	3.32	3.21	3.73	3.34	3.85	4.08	4.06	3.63
Metasoma I W	3.23	3.15	3.56	2.83	4.32	4.31	4.43	4.42
Metasoma I H	2.52	2.47	2.71	2.41	3.27	3.10	3.31	3.28
Metasoma II L	3.78	3.63	4.38	3.68	4.38	4.55	4.27	4.77
Metasoma II W	3.19	3.10	3.53	3.14	4.21	4.48	4.08	4.16
Metasoma II H	2.56	2.50	2.74	2.27	3.23	3.09	3.25	3.39
Metasoma III L	3.90	3.93	4.44	3.50	4.97	4.75	4.69	4.97
Metasoma III W	3.11	3.05	3.54	2.70	4.14	3.88	4.23	4.04
Metasoma III H	2.56	2.51	2.76	2.33	3.18	3.14	3.21	3.39
Metasoma IV L	4.82	4.43	5.29	4.58	5.49	5.72	5.35	5.53
Metasoma IV W	3.03	2.84	3.35	2.70	3.84	3.52	3.77	3.94
Metasoma IV H	2.59	2.51	2.96	2.32	3.21	3.49	3.34	3.39
Metasoma V L	6.69	6.33	7.27	6.32	7.95	7.98	7.90	8.34
Metasoma V W	2.87	2.76	3.28	2.44	3.60	3.32	3.54	3.66
Metasoma V H	2.40	2.46	2.64	2.00	3.16	2.82	3.23	3.08
Telson L	6.85	6.50	7.25	6.74	8.65	8.02	8.34	8.43
Vesicle L	4.63	4.57	4.62	4.15	5.99	5.72	5.50	6.04
Vesicle W	2.87	2.73	3.06	2.76	3.86	3.51	3.77	4.17
Vesicle H	2.16	2.09	2.47	2.04	2.90	2.77	2.98	2.83
Aculeus L	2.53	2.72	2.45	2.38	2.71	3.02	2.63	3.32
Pedipalp L	20.48	20.06	20.50	18.48	24.96	24.22	24.30	25.63
Pedipalp femur L	4.80	4.75	5.30	4.16	5.75	5.79	5.73	6.14
Pedipalp femur W	1.81	1.69	1.81	1.59	2.19	2.06	2.37	2.43
Pedipalp femur H	1.26	1.21	1.41	1.16	1.65	1.59	1.67	1.81
Pedipalp patella L	4.72	4.68	5.05	4.51	6.18	5.92	6.11	6.07
Pedipalp patella W	2.23	2.14	2.27	1.88	2.71	2.60	2.73	2.87
Pedipalp patella H	2.10	2.02	2.08	1.83	2.37	2.22	2.73	2.59
Pedipalp Chela L	9.53	9.45	9.91	8.88	11.47	11.21	11.50	12.08
Pedipalp Manus W	4.66	4.48	4.59	4.15	5.33	5.05	4.99	5.60
Pedipalp Manus T	3.18	3.01	3.35	2.93	3.85	3.65	3.69	3.85
Chela Finger fixed L	3.45	3.96	3.81	3.39	4.40	4.80	4.91	4.73
Chela finger movable L	6.27	6.35	6.21	6.01	7.88	7.46	7.52	7.90
Pectine L	5.48	5.67	6.16	5.13	5.09	5.38	5.39	4.75
Pectine W	1.42	1.43	1.91	1.55	1.01	1.18	1.24	1.06

All specimens included in the description of this species were found by blacklight on 30 May 2021 and additional specimens were found by users of iNaturalist.org on 19 May 2021 and 1 March 2022. *Paruroctonussoda* sp. nov. was abundant at all localities where it was found. We found a higher density of surface-active adult males than surface-active adult females, and a higher density of surface-active adults than surface-active juveniles. A single late-instar juvenile female *Paruroctonussoda* sp. nov. was found to be whitish in color and completely lacked fuscousity (Fig. 13), possibly indicating hypomelanism. It is unclear whether this was a low-probability chance event or if there is a significant portion of the population of *Paruroctonussoda* sp. nov. with this trait. A gravid adult female collected and maintained alive in captivity gave birth in mid-August to 51 offspring, of which all except one survived until the first molt (Fig. 14).

##### Conservation.

Fortunately, the entirety of the range of *Paruroctonussoda* sp. nov. is encompassed within the Carrizo Plain National Monument, rendering the species safe from the primary anthropogenic threats to scorpions: land alteration and habitat destruction due to human development.

##### Etymology.

*Paruroctonussoda* sp. nov. is named after Soda Lake, which is the only locality this species is known from. The name also reflects the highly alkaline soils this species inhabits.

#### ﻿ Paruroctonus conclusus sp. nov.

Taxon classificationAnimaliaScorpionesVaejovidae

F954A2E6-D551-5416-8984-72B0EDEEE809

https://zoobank.org/3C830C30-4F3E-400B-9661-046EB7726D1F

Figs 15 , 16 , 17 , 18 , 19 , 20 , 21 , 22 , 23 , 24 , 25 , 26 , 27 , Table 2

##### Type material.

***Holotype***: USA • 1 ♂; California, Kern County, southeastern edge of Koehn Lake; 35.3123, -117.8614; 581 m a.s.l.; 3 July 2021; collector leg Prakrit Jain; collected at night using handheld UV light; CASENT 9101936.

***Paratypes*.** USA • 4 ♂, 1 ♀; California, Kern County, southeastern edge of Koehn Lake; 35.3123, -117.8614; 581 m a.s.l.; 3 July 2021; collector leg Prakrit Jain; collected at night using handheld UV light; CASENT 9101937.

USA• 1♀; California, Kern County, southeastern edge of Koehn Lake; 35.3123, -117.8614; 581 m a.s.l.; 2 August 2021; collector leg Harper Forbes; collected at night using handheld UV light; CASENT 9101938.

##### Diagnosis.

Differs from other *Paruroctonus* species found in the Northwestern Mojave Desert and its surrounding mountains (The Tehachapis, the southern Sierra Nevada, and the northeastern Transverse Range) by a combination of the following characteristics: 1: Fuscous markings entirely absent from the metasoma and pedipalps and heavily reduced to absent from the carapace and tergites (Figs 15, 16, 18, 20). 2: Chelal fingers deeply scalloped in adult males, leaving a large proximal gap when closed. 3: Macrosetae on the metasomal lateral supramedian and ventral submedian carinae of metasomal segments I–IV follow the patterns 0,1,1,2 and 2,2,2,3, respectively (Fig. 23). 4: Presence of only a single large retrolateral median macroseta on the pedipalp patella (between the *em_1_* and *est* trichobothria). 5: The number of primary denticles on the fixed and movable fingers, excluding the proximal row, 31–36 and 42–51, respectively. 6: Chela length / Manus width ratio 2.20–2.4 in adult males and 2.46–2.52 in adult females. 7: Mid-retrosuperior macroseta always present on basitarsus II. 8: Chelal dorsomedian carina strong and smooth on its distal half, curving prolaterally between the *db* and *dsb* trichobothria (Fig. 20). 9: Prolateral ventral macroseta absent on movable finger of the chela (Fig. 20).

Comparisons are provided for the four other *Paruroctonus* found in the Northwestern Mojave desert and its surrounding mountains (The Tehachapis, the southern Sierra Nevada, and the northeastern Transverse Range), *P.becki*, *P.marksi*, *P.boreus*, and *P.silvestrii*. Of these, only *P.becki* is found in sympatry with *P.conclusus* sp. nov. The other three are found at a considerable distance away in very different habitats: sand dunes for *P.marksi*; grassland or chaparral for *P.silvestrii*; and high desert, scrubland, or conifer woodland, typically well above 800 m elevation, for *P.boreus* (especially in the Mojave Desert and surrounding regions). *P.becki* can be easily differentiated by its significantly more slender chela. Morphological comparisons are also provided for the other species described in this paper, *Paruroctonussoda* sp. nov., but the two species can be easily separated by range. Other *Paruroctonus* which may have certain morphological similarities to *P.conlcusus* sp. nov. are entirely allopatric.

*P.becki* differs from *P.conclusus* sp. nov. in the following characters relating to the numeration in the above diagnosis: (2) Chelal fingers not scalloped (straight), leaving no proximal gap when closed. (3) Metasomal macrosetae on the lateral supramedian and ventral submedian carinae of segments I–IV follow the patterns 0,2,3,2–3 and 3,4,4–5,4–5, respectively. (4) Presence of 4–6 retrolateral median macrosetae on the pedipalp patella. (5) Primary denticles 46–50 on the fixed finger and 61–66 on the movable finger. (6) Chela length/manus width ratio 3.40–3.51 in males, 3.44–3.56 in females.

*P.marksi* differs from *P.conclusus* sp. nov. in the following characters relating to the numeration in the above diagnosis: (2) Chelal fingers in males moderately scalloped, leaving a small gap when closed. (3) Metasomal macrosetae on the lateral supramedian and ventral submedian carinae of segments I–IV follow the patterns 0,1,1,2 and 2–3,3–5,3–4,4–5, respectively (Haradon 1984a). (7) Mid-retrosuperior macroseta always absent on basitarsus II (Haradon 1984a). (8) Chelal dorsomedian carina weakly crenulate and irregular on its distal half and terminates near the *db* trichobothria without curving (Haradon 1984a).

*P.boreus* differs from *P.conclusus* sp. nov. in the following characters relating to the numeration in the above diagnosis: (1) Heavy fuscous markings present on the tergites, carapace, and the ventral surface of the metasoma, especially on segments II–IV. (3) Macrosetae on the ventral submedian carinae of metasomal segments I–IV follow the pattern 2,2–3,3,3 (rarely 2,2,2,3). (5) The number of primary denticles on the fixed finger, excluding the proximal row, 35–46 (37–52 according to Haradon 1985). (8) Chelal dorsal median carina continues to be weakly crenulate and irregular on its distal half and ends near the *db* trichobothria without curving. (9) A single prolateral ventral macroseta is typically present on the proximal half of the movable finger of the chela. Of these characters, (3), (5) and (9) may overlap with *P.conclusus* sp. nov., however, overlap is rare and ambiguity in all 3 characters is highly unlikely.

*P.silvestrii* differs from *P.conclusus* sp. nov. in the following characters relating to the numeration in the above diagnosis: (1) Heavy fuscous markings present on the tergites, carapace, pedipalps, and the ventral surface of the metasoma. (2) Chelal fingers not scalloped (straight), leaving no proximal gap when closed. (3) Macrosetae on the ventral submedian carinae of metasomal segments I–IV follow the pattern 2–3,3,3–4,3–4. (4) Presence of 2–4 retrolateral median macrosetae on the pedipalp patella. (5) Primary denticles 41–52 on the fixed finger and 54–68 on the movable finger. (6) Chela length/manus width ratio 2.59–2.70 in males, 2.75–3.06 in females. (8) Chelal dorsal median carina weakly crenulate to smooth and weak on its distal half and ends near the db trichobothria without curving. (9) Two prolateral ventral macrosetae present on the movable finger of the chela.

*P.soda* sp. nov. differs from *P.conclusus* sp. nov. in the following characters relating to the numeration in the above diagnosis: (1) Significant fuscous markings present on the carapace and tergites. (3) Macrosetae on the ventral submedian carinae of metasomal segments I–IV follow the pattern 1–2,2,2,2. (4) No large retromedian macrosetae present on the pedipalp patella.

##### Description of male holotype.

***Coloration* (Figs 15–17).** Carapace pale yellow anteriorly to tan posteriorly. Very faint fuscous markings restricted to the posterior extent of the interocular triangle. Tergites mostly brown with lighter, yellowish posterior and lateral margins. Fuscosity on the tergites extremely indistinct, almost absent. Legs whitish to pale cream. Pedipalps light tan with darker carinae and orangish fingers. Metasoma tan, with slightly darker carinae. Telson pale yellow, base of aculeus dark reddish-brown, and aculeus black. Sternites dark brown with brown spiracles. Pectines, sternum, and genital operculum tan to pale yellow.

***Carapace* (Figs 18, 19).** Anterior margin roughly straight to very slightly concave with three pairs of distinct macrosetae. Large granules present sparsely and irregularly; very fine and evenly spaced granules present between the large granules. The largest granules are clustered in the posterior median portion of the carapace, and large granules decrease in size anteriorly and laterally. Posterior, lateral, and anterior margins finely crenulate. Posterior median sulcus narrow and moderately deep, with some posterior granulation. Anterior median and median ocular sulci shallow, free of large granules. Lateral ocular and posterior lateral sulci broad and shallow, free of large granules. Central lateral sulcus broad and shallow, with sparse granules. Interocular region of the carapace smooth with sparse granules anteriorly. Median ocelli separated by a distance greater than the width of one ocellus. 2/3 lateral ocelli present on each side. Single pairs of macrosetae present posterior to the median ocelli, situated between the lateral ocelli and the margin of the carapace, and roughly halfway between the posterior median sulcus and the posterior margin of the carapace, in line with the posterior edge of the ocular tubercle.

***Mesosoma* (Figs 16–18).** Tergites I–VI smooth to very finely granular, except on the posterior-lateral half of each side, which is smooth (tergite I) to granular (tergite VI), and the posterior margin, which is ranges from smooth (tergite I) to weakly and finely granular (tergite VI). Median longitudinal carina weak, smooth on I and weakly irregularly crenulate to irregularly crenulate on II–VI. Submedian longitudinal sulci indistinct. One pair of small posterior sub-median setae on tergites I–V, vestigial on VI. Tergite VII essentially smooth anteriorly and posteriorly, lateral-median areas sparsely granular. Lateral marginal carina finely crenulate; dorsolateral and dorsal sub-median carinae strongly crenulate. Median longitudinal carina weakly crenulate. Sternites III–VI sparsely setose and smooth. Sternite VII smooth anteriorly and finely granular laterally, with ventral submedian carinae indistinct and very weakly crenulate and lateral marginal carinae finely crenulate.

***Genital operculum* (Fig. 19).** Sclerites roughly triangular with rounded corners, ca. as wide as long. Overlapping medially and separated slightly only at the posterior edge, with protruding genital papillae. Several macrosetae present on each sclerite.

***Sternum* (Fig. 19).** Type 2 with posterior emargination absent, apex deep, slightly wider than long, smooth except very finely granular along the slopes of the apex. Three pairs of macrosetae.

***Pectines* (Fig. 19).** Long, thin, and densely hirsute, with 25/26 tightly packed teeth on each side. Middle lamellae roughly circular distally, highly irregular in size and shape proximally; roughly 21/22 distinct and separated sclerotized sections are visible under ultraviolet illumination.

***Legs*. *Carinae*.** Retroventral carina on leg I femur finely crenulate and nonlinear; linear on subsequent legs. Superior carina on leg I femur weakly and finely crenulate, decreasingly distinct on subsequent legs. Proventral carina sparsely, finely, and weakly crenulate on leg I patella, decreasingly distinct on subsequent legs and nearly absent by leg IV. Intercarinal spaces on legs smooth with occasional sparse, fine granules on the femur.

***Telotarsi*.** Telotarsal retroinferior terminal macrosetae on legs I–IV 1/1, 1/2, 2/2, 2/2; other telotarsal retroinferior macrosetae on the distal half of telotarsi I–IV 1/1, 1/1, 2/2, 2/2. Two telotarsal retromedial macrosetae on each leg, with one always at the retromedial terminal position. Two large telotarsal retrosuperior macrosetae on each leg with consistent positions. Single proinferior terminal macroseta on each leg except two on dextral leg II. Single proinferior distal macroseta on each leg, single other proinferior macroseta on legs II–IV. Two telotarsal promedial macrosetae on legs I–II at terminal and distal positions; one on legs III–IV in terminal position. Two large telotarsal prosuperior macrosetae on each leg in terminal and medial positions. Telotarsal superior macrosetae on legs I–IV 1/1, 1/1, 1/0, 0/0. Single telotarsal superioterminal macroseta present on all legs.

***Basitarsi*.** Three basitarsal spine rows present on legs I and II; proventral and retroventral spine rows equally dense and retrosuperior spine row less dense. The retroventral spine row extends ca. three-fourths the entire length of the segment, the proventral spine row extends through ca. half the segment, and the retrosuperior spine row extends irregularly through around half. On leg III, the proventral spine row is absent and the retrosuperior and retroventral spine rows are heavily reduced in density. On leg IV, both the proventral and retroventral spine rows are absent and the retrosuperior spine row is heavily reduced in density, nearly absent. Basitarsal retroventral macrosetae on legs I–IV follow the pattern 2/3, 4/5, 6/6, 4/4 (excluding the distal retroventral spinoid macroseta at the terminus of the retroventral spine row), with variably sized setae. Spinoid basitarsal proventral macrosetal pattern on legs I–IV is 2/2, 2/2, 2/2, 3/3; an additional thinner terminal ventral macroseta is present on legs II–IV. Superior basitarsal macrosetae on legs I–IV consist of two spinoid macrosetae at the distal and mid retrosuperior positions; two macrosetae at the distal and mid prosuperior positions, except leg IV which has only the distal prolateral macroseta; one macroseta at the distal superiomedian position adjacent to the distal retrosuperior macroseta on legs I–III; and large superiomedian macrosetae following the pattern 5/5, 5/5, 5/5, 4/5. Prolateral macrosetae on legs I–IV, excluding one on the margin, follow the pattern 3/3, 3/3, 3/3, 2/2.

***Pedipalps* (Figs 20–22). *Femur*.** Dorsal prolateral carina crenulate with two macrosetae on the proximal half; dorsal retrolateral carina also crenulate with two macrosetae on the proximal three-fourths. Dorsal surface sparsely granular. Retrolateral dorsosubmedian carina weakly crenulate with 3/4 median macrosetae and an additional one on the distal margin; retrolateral surface otherwise smooth aside from a few proximal granules. One additional large macroseta in a distal inframedian position on the retrolateral surface. Three small ventral retrolateral macrosetae present. Ventral retrosubmedian carinae vestigial, irregularly granular with granules decreasing in size distally. Ventral prolateral carina irregularly crenulate. Prolateral surface irregularly granular with three prolateral ventral macrosetae on the proximal two-thirds including one on the proximal marginal carina, one prolateral ventrosubmedian macroseta near the midpoint, and a pair of macrosetae on the distal margin.

***Patella*.** Dorsal retrolateral carina weakly crenulate with a proximal macroseta; dorsal prolateral carina crenulate, also with a proximal macroseta. Dorsal surface smooth. Retrolateral median carinae indistinct and very weakly crenulate, retrolateral surface otherwise smooth. A single median and two distal macrosetae are present on the retrolateral surface. Ventral retrosubmedian carina weakly crenulate with a distal macroseta; ventral prolateral carina crenulate, also with a distal macroseta. A proximal macroseta is present at the junction of the ventral retrosubmedian carina and the finely crenulate ventral median carina. Ventral surface smooth. Prolateral median carina indistinct, represented by a few large granules. Prolateral surface otherwise smooth. Prolateral surface with large proximal supramedian, proximal inframedian, distal inframedian, and distal supramedian macrosetae.

***Chela*.** Dorsal prolateral carina indistinct, non-linear, and crenulate on the manus with a medial macroseta. Dorsal median carina weakly crenulate proximally and smooth distally, curving prolaterally between the *db* and *dsb* trichobothria and terminating at the dorsal prolateral carina. A single macroseta is present at the proximal terminus of the dorsal median carina. Dorsal retrosubmedian carina vestigial, consisting of only a few weak granules, and extending through less than the proximal fifth of the manus. Dorsal retrosubmedian accessory carina also vestigial, extending through less than the proximal tenth of the manus, with a proximal macroseta. Dorsal retrolateral carina very weakly crenulate proximally and smooth distally with a medial and distal macroseta on the manus. Retrolateral median carina very weakly granular and unpigmented, with a single medial macroseta. Ventral retrolateral carina irregular and weakly crenulate, with 1/0 proximal and three non-linear medial macrosetae. Intercarinal spaces on the dorsal and retrolateral surfaces smooth aside from occasional sparse granules. Ventral prosubmedian carina irregular and weakly crenulate, with a one proximal and one medial macroseta. Ventral surface mostly smooth with some distal granulation. Prolateral ventral carina crenulate to weakly crenulate with a proximal and distal macroseta. Prolateral median carina crenulate to weakly crenulate with a proximal and medial macroseta. Two further small carinae are present near the base of the fixed finger, both of which are evenly and finely crenulate. Prolateral surface of the manus otherwise mostly smooth with some weak and irregular granulation in the distal half. The fingers are heavily scalloped, leaving a large proximal gap when closed. The chela is uniformly finely granular at the base of this gap. Retrolaterally and prolaterally, the fingers are smooth except some fine proximal granulation. 18/21 small macrosetae and numerous microsetae are present on the ventral surface of the movable finger. No prolateral ventrolateral macrosetae are present on the movable finger. The movable finger has one proximal and one medial prolateral median macroseta and one proximal retrolateral median macroseta. No proximal prolateral ventral macroseta is present on the movable finger. The fixed finger has one prolateral medial macroseta and one proximal prolateral dorsolateral macroseta. The fixed finger has one retrolateral medial and one distal dorsal retrolateral macroseta. Both the fixed and movable fingers have five retrolateral enlarged denticles dividing the primary denticles into six sub-rows, with an additional retrolateral enlarged denticle at the distal extent of the movable finger, alongside the distal hook. On the fixed finger, rows I–VI contain 5/3, 6/4, 6/7, 7/8, 10/10, 10/11 primary denticles with a total row I–V count of 34/33. On the movable finger, rows I–VI contain 6/5, 7/8, 10/9, 10/10, 11/13, 8/8 primary denticles with a total row I–V count of 44/45. Each retrolateral enlarged denticle as well as the distal finger-tip hook is accompanied by a single prolateral supernumerary denticle, for a total of six on the fixed finger and seven on the movable finger. There is a single macroseta posterior to each supernumerary denticle with the exception of the two most distal ones on each finger. Two further macrosetae are present near the proximal to the most proximal primary denticle on the fixed finger.

***Metasoma* (Fig. 23).** Dorsal surface I–V smooth. Dorsolateral carinae on segments I–IV strongly crenulate to serrate, weakly crenulate on V. Lateral supramedian surface essentially smooth with a few scattered granules. Lateral supramedian carinae I–IV strongly crenulate to serrate. Lateral surface smooth. Lateral inframedian carinae crenulate on I–III, extending through only the posterior fifth of segments II–III. Lateral median carinae indistinct and weakly crenulate on V, extending ca. a third of the way up the segment. Ventrolateral carinae I–IV smooth, becoming weakly crenulate on the posterior fifth of segment III and the posterior half of segment IV. Ventrolateral carinae on segment V strongly crenulate to serrate. Ventral surface of segment I–IV smooth; ventral surface sparsely granular on segment V. Ventral sub-median carinae on I–IV smooth and unpigmented, indistinct on I. Ventromedian carinae on segment V are crenulate and irregular. Dorsolateral macrosetae I–IV follow the pattern 0,1,1,2. Four dorsolateral macrosetae on V. Lateral supramedian macrosetae I–IV follow the pattern 0,1,1,2. Two Lateral median macroseta on V. Lateral inframedian macrosetae I–III follow the pattern 1,0,0. Ventrolateral macrosetae I–V, excluding any on the posterior margin of the segment, follow the pattern 2,3,3,3. Six ventrolateral macrosetae on V excluding any on the posterior margin. Ventral submedian macrosetae I–IV, excluding those on the posterior margin of the segment, follow the pattern 2,2,2,3. Four pairs of macrosetae are present between the ventromedian and ventrolateral carinae on segment V. Two pairs of macrosetae on the ventral posterior margin of metasomal segments IV and V; a single pair of macrosetae on the ventral posterior margins of other metasomal segments.

***Telson* (Fig. 23).** Very weakly granular on the ventral anterior portion, otherwise smooth. Sparsely setose ventrally and laterally.

***Hemispermatophore* (Fig. 24).** Hemispermatophore decreasing in width from pedicel to stalk, three-fold bauplan (Monod et al. 2017). Stalk wide, relatively straight, and dorso-ventrally flattened. Distal carina and lamellar hook sclerotized, lamellar hook prominent and weakly bifurcate at terminus. Mating plug weakly sclerotized, moderate in size with a single lobed base and long stem terminating in a prominent barb.

**Female.** Larger carapace in comparison to the male. Carapace smoother, with essentially smooth interocular triangle and very weak granulation in the posterior-lateral areas. Tergites also smooth, with granulation largely restricted to the posterior and lateral margins and lateral fifth or less on each side. Chela less incrassate, with fingers not scalloped, leaving a negligible gap when closed. Most proximal row on the chelal fixed finger with 16–19 primary denticles; most proximal row on the chelal movable finger with 8–10 primary denticles. Metasoma more robust than in males. Pectines smaller overall with fewer teeth, 16–19; middle lamellae slightly more regular than in males; 13–15 separated and sclerotized sections visible under ultraviolet illumination. Genital operculum sclerites do not overlap, and are slightly separated through their entire length.

**Variation. *Coloration* (Figs 15–17).** Coloration largely constant, with pale yellow-brown carapace, pedipalps, and metasoma; lighter legs; and a darker mesosoma. Fuscousity very faint, restricted to the edges of the interocular triangle, the extreme posterior-lateral corners of the carapace, and the anterior portion of the tergites.

***Carapace* (Figs 18, 19).** Density and distribution of granulation variable. Highest density of large granulation is found in the posterior median section. Posterior-lateral margins with moderate to very weak granulation; interocular triangle with weak to essentially absent granulation. Lateral eyes typically three on each side.

***Mesosoma* (Figs 16–18).** Tergites with variable amounts of granulation. Margins granular to weakly granular, posterior and lateral portions granular to smooth. Small posterior submedian setae sometimes absent on VI.

***Pectines* (Fig. 19).** Males with 23–26 teeth. Roughly 18/23 distinct and separated sclerotized middle lamellae are visible under ultraviolet illumination

***Legs*.** Telotarsal setation somewhat variable. Retroinferior terminal and other retroinferior macrosetae on the distal half of telotarsi I–IV both within the ranges 1,1–2,2,2. Typically two retrosuperior and two retromedial macrosetae, with an additional large macroseta rarely present on legs III–IV. One or multiple extra small retrosuperior or retromedian macrosetae occasionally present on any leg. Telotarsal proinferior and prosuperior macrosetae consistent with occasional asymmetric additions or deletions. Promedian macrosetae on legs I–IV within the ranges 2–3,2,1–2,1–2. Superior median macroseta on legs I–IV within the ranges 1,1,0–1,0–1, with variation in size. Basitarsal setation highly variable. Retroventral macrosetae on legs I–IV, excluding only the one on the distal margin, within the ranges 2–4,4–7,6–7,4–6. Proventral macrosetae on legs I–IV, excluding the thinner proventral terminal macroseta on legs II–IV, within the ranges 2,2–3,2–3,3. Spinoid retrosuperior macrosetae always present in the mid and distal positions. Prosuperior macrosetae typically present at the mid and distal positions on legs I–III and at the distal position on leg IV but one or both may be present, absent, or accompanied by an additional prosuperior macroseta on any leg. Distal superiomedian macroseta typically adjacent to the distal retrosuperior macroseta but variable in position and occasionally absent on any leg. Large retrosuperior setae excluding the aforementioned retrosuperior, prosuperior, and distal superiomedian seta typically consist of three distal and two proximal ones for a total of five on legs I–III and two or three distal and two proximal ones for a total of four or five on leg IV; however, an additional large macroseta may be present on legs I–III and additional small macrosetae may be present on all legs. Larger prolateral macrosetae on legs I–IV variable and non-linear, within the ranges 3,3–4,3–4,2–3; typically three on each.

***Pedipalps* (Figs 20–22).** Femur with 3–5 large retrolateral dorsosubmedian macrosetae and an occasionally present small distal dorsal retrolateral macroseta. Other macrosetae on the pedipalp femur and patella consistent apart from occasional asymmetrical deletions, which are most frequent on the patella retrolateral median macroseta. On the chela, most macrosetae consistent. An additional ventral retrolateral for a total of four and an additional retrolateral median macroseta for a total of two occasionally present. Asymmetrical deletions sometimes occur on most macrosetae on the manus. Fixed finger retrolateral median macroseta sometimes missing. Movable finger with 17–21 ventral macrosetae. Number of primary denticles in rows I–V on the fixed finger within the ranges 2–5, 4–7, 6–8, 7–10, 9–12. Number of primary denticle in row VI on the fixed finger of males 7–11. Number of primary denticles in rows I–VI on the movable finger within the ranges 5–8, 7–9, 8–10, 9–10, 11–16. Number of primary denticle in row VI on the movable finger of males 5–10. Total number of primary denticles on rows I–V on the fixed and movable fingers 31–36 and 42–51, respectively with no obvious sexual dimorphism.

***Metasoma* (Fig. 23).** Dorsolateral macrosetae on I–IV, lateral superiomedian macrosetae on I–IV, lateral inframedian macrosetae on I–IV, ventral submedian macrosetae on I–IV, posterior marginal macrosetae on I–IV, and macrosetae on V consistent. Ventrolateral macrosetae on I–IV follow the pattern 2–3,2–3,3,3. Occasional asymmetrical macrosetal deletions on dorsal and lateral surfaces; frequent asymmetrical macrosetal deletions on ventral surface.

##### Remarks.

The most valuable taxonomic characters for *P.conclusus* sp. nov. are:

1. The lack of fuscousity on the carapace and tergites is very consistent and is reliably different than in certain other *Paruroctonus*.
2. The macrosetal patterns on the pedipalps and metasoma are mostly consistent and provide an excellent diagnostic against many other *Paruroctonus*.

Other taxonomic characters which may be valuable in some cases, but are typically not useful, include:

1. The telotarsal macrosetae are somewhat variable and have different counts than certain other *Paruroctonus*.
2. The granulation on the carapace and tergites is fairly variable but is notably different from certain other *Paruroctonus*. This character, however, can be difficult to quantify.
3. The basitarsal macrosetae are generally extremely variable and are only helpful for differentiating *P.conclusus* sp. nov. from certain psammophilous *Paruroctonus*. The basitarsal spinoid distal and mid retrosuperior macrosetae are not variable but are still only helpful for differentiating *P.conclusus* sp. nov. from these psammophiles.
4. The granulation on the pedipalps, legs, and metasoma is somewhat variable and difficult to quantify. It is fairly similar to that of most other *Paruroctonus* species, although in isolated examples may be used for diagnosis.
5. The morphometric ratios of different aspects of the metasomal segments and chela are typically fairly consistent but overlap with those of many other *Paruroctonus*.
6. The pectinal tooth counts are somewhat variable and overlap with those of most other *Paruroctonus*. Middle lamellae counts are also not taxonomically valuable, as they are typically ambiguous.
7. The chelal primary denticle counts are somewhat variable and overlap with those of most other *Paruroctonus*.

##### Habitat, distribution, and ecological notes.

*Paruroctonusconclusus* sp. nov. is known from only a single locality along the edge of Koehn Lake, which is located within Kern County, California (Fig. 25). Koehn lake is an ephemeral, alkaline desert lakebed at the center of the Fremont Valley in the northwestern Mojave Desert. This valley is bounded on the north by the El Paso mountains and on the south by the Rand mountains, resulting in it being an endorheic basin draining primarily into its lowest point, Koehn Lake, which lies at ca. 570 m a.s.l. (RWMG 2019).

Over the past million years, water levels in Mojave Desert lakes have varied significantly, with several periods of increased moisture where Koehn lake, with other lakes in the Mojave, expanded in size and filled with perennial water and other periods where these lakes shrunk and dried up (Stoffer 2004). Since the end of the most recent Pleistocene ice age, these lakes have generally decreased in size with some minor fluctuation (Stoffer 2004). Currently, playas throughout the Mojave Desert only occasionally hold water (Enzel et al. 1992). Marsh-specialist flora and fauna species distributions typically increase and decrease in area with the surface area of their associated lakes (Stoffer 2004), so we hypothesize that the distribution of *P.conclusus* sp. nov. was historically more extensive than it is currently and that it has shrunk with a drying climate.

The Fremont Valley region is typical of the Mojave desert with characteristic low levels of precipitation concentrated in the winter months, around 15 cm annually, and high summer temperatures, typically in excess of 35 °C (RWMG 2019). This results in an overall arid desert climate with relatively more moisture concentrated at and around Koehn Lake.

The type locality of *Paruroctonusconclusus* sp. nov. is on the southeast edge of this lakebed in an area of increased moisture (Fig. 25). *P.conclusus* sp. nov. was found at the type locality on two moonless summer nights in 2021, with a moderate level of surface activity on July 3 and a low level of surface activity on August 2. Both dominant plant species found at the type locality, *Allenrolfeaoccidentalis* and *Suaedanigra*, are alkali sink specialists (Munz and Keck 1949). Correspondingly, the soil at the type locality is mostly clay, although in a few spots, it is covered with a thin layer of sand. *P.conclusus* sp. nov. does not appear to prefer either the open clay or the sand-covered clay over the other. Several *P.conclusus* sp. nov. were seen partially or fully concealed within burrows or cracks in the clay soil, indicating that they are a largely fossorial species. We hypothesize that the increased moisture and softer clay-rich soil facilitate burrowing and are the primary factors restricting *P.conclusus* sp. nov. to the lakeside alkali-sink habitat (Fig. 26).

*P.conclusus* sp. nov. is sympatric with three other scorpion species: *Hadrurusarizonensis* Ewing 1928, *Paravaejovisconfusus* (Stahnke, 1940), and *Paruroctonusbecki*. The former two can be found throughout the desert flats habitat surrounding the alkali-sink area adjacent to the Koehn lakebed; however, *P.becki* was only observed immediately adjacent to the lakebed, in sympatry with *P.conclusus* sp. nov. We conducted significant additional sampling at three other localities around Koehn Lake: the southernmost point, the northernmost point, and the northwestern corner. Suitable habitat, which is dominated by *A.occidentalis* and *S.nigra*, was not found at the former two locations. The northwestern corner of the lakebed had a small area of seemingly suitable habitat, and while *P.becki* was found to be surface-active in high density, no *P.conclusus* sp. nov. were observed despite significant sampling effort. While more sampling is necessary to make a high-confidence determination of absence, we currently believe that it is unlikely *P.conclusus* sp. nov. is found at the northwestern edge of Koehn Lake. Another locality where the habitat appears to be potentially suitable for *P.conclusus* sp. nov. based on satellite imagery exists along the western edge of the lakebed; however, we were unable to sample it due to it being on privately-owned land.

Predation by *P.conclusus* sp. nov. was recorded once, by an adult male on an adult *Paruroctonusbecki*. This indicates that these two species exist at least partially in microsympatry.

##### Conservation.

*Paruroctonusconclusus* sp. nov. has one of the smallest known distributions of any species of *Paruroctonus*, existing in a stretch of suitable habitat only a couple kilometers in length and no more than a few hundred meters wide. This limited range makes it especially susceptible to extinction. Both primary threats to this scorpion are anthropogenic in origin or extent: destruction of habitat and alterations in climate. The Fremont Valley region of California was home to ca. 20,800 residents in 2020, a number that is projected to grow to 29,400 over the next 20 years (RWMG 2019). This will cause further degradation of the land, not only in the form of housing development but also due to water extraction, electricity production, and other economic activity. The small community of Cantil abuts Koehn Lake on its western shore and contains some agricultural activity. The formerly inhabited town of Saltdale is at the northern extent of Koehn Lake, from where it historically mined valuable salts from the lakebed. While the mine is currently not operational, the entirety of Koehn Lake remains open to the potential of mining (BLM 2005). Another major industry in the Fremont Valley is solar electricity production (RWMG 2019). Two operational farms, owned by Beacon Solar and Springbok, are located in close proximity to Koehn Lake (RWMG 2019). These factors significantly threaten the habitat of *Paruroctonusconclusus* sp. nov., not only by direct habitat alteration but also by indirect downstream effects such as production of waste products, usage of groundwater, and possible alterations to the region’s hydrology. Agriculture and mining use large amounts of water, and solar farms can have large-scale destructive effects on desert ecosystems.

**Table 2.** Table of measurements of 5 adult male and 2 adult female *Paruroctonusconclusus* sp. nov., in mm.

	Holotype male	Paratype male	Paratype male	Paratype male	Paratype male	Paratype female	Paratype female
CASENT #	9101936	9101937	9101937	9101937	9101937	9101938	9101937
Total L	43.29	38.11	40.96	36.87	38.91	41.09	43.68
Carapace L	5.32	4.56	5.04	4.35	4.94	5.82	5.88
Prosoma posterior W	4.69	4.12	4.47	4.10	4.45	5.52	5.47
Prosoma median W	4.13	3.28	3.67	3.23	3.50	4.20	4.70
Mesosoma L	10.98	8.65	9.07	9.33	10.02	11.28	10.74
Metasoma L	28.13	24.38	26.74	23.56	25.03	26.35	27.35
Metasoma I L	3.09	2.88	3.04	2.53	2.94	2.73	2.86
Metasoma I W	2.73	2.30	2.49	2.37	2.66	2.89	2.81
Metasoma I H	2.08	1.95	2.08	1.71	2.05	2.11	2.44
Metasoma II L	3.53	3.18	3.47	3.00	3.72	3.61	3.41
Metasoma II W	2.81	2.24	2.50	2.46	2.60	2.76	2.72
Metasoma II H	2.18	1.97	2.00	1.76	1.97	2.31	2.41
Metasoma III L	3.90	3.42	3.66	3.28	3.65	3.45	3.74
Metasoma III W	2.74	2.06	2.21	2.25	2.38	2.75	2.60
Metasoma III H	2.20	1.98	1.97	1.77	1.95	2.16	2.22
Metasoma IV L	4.65	4.19	4.53	3.94	4.44	4.29	4.42
Metasoma IV W	2.40	2.08	2.17	2.05	2.19	2.70	2.43
Metasoma IV H	2.28	1.94	2.08	1.71	1.98	2.38	2.24
Metasoma V L	6.75	5.83	6.46	5.70	6.23	6.09	6.57
Metasoma V W	2.36	1.92	2.12	1.87	2.07	2.47	2.57
Metasoma V H	2.13	1.88	1.92	1.70	1.77	2.40	2.31
Telson L	5.79	5.37	5.87	5.34	5.38	6.60	6.63
Telson vesicle L	4.32	3.60	3.96	3.68	3.54	4.12	4.26
Telson vesicle W	2.32	1.95	2.16	1.80	2.03	2.44	2.49
Telson vesicle H	1.87	1.51	1.63	1.44	1.56	2.08	2.13
Telson aculeus L	1.86	1.73	1.89	1.70	1.75	2.07	2.28
Pedipalp L	18.35	16.28	17.87	15.80	16.88	19.48	19.51
Pedipalp femur L	4.56	4.08	4.25	3.80	4.08	4.54	4.38
Pedipalp femur W	1.41	1.23	1.31	1.23	1.39	1.51	1.65
Pedipalp femur H	0.95	0.90	0.94	0.83	0.90	1.01	1.12
Pedipalp patella L	5.14	4.20	4.35	3.69	4.64	4.92	4.92
Pedipalp patella W	1.71	1.42	1.50	1.37	1.54	1.75	1.91
Pedipalp patella H	1.67	1.47	1.56	1.33	1.50	1.78	1.87
Pedipalp chela L	8.52	7.51	8.25	7.14	7.53	8.95	9.21
Pedipalp manus W	3.83	3.11	3.50	3.12	3.43	3.64	3.65
Pedipalp manus T	2.80	2.05	2.42	2.16	2.42	2.51	2.41
Chela finger fixed L	3.45	3.13	3.22	2.78	2.84	3.53	3.96
Chela finger movable L	5.26	4.79	5.41	4.61	4.56	5.56	5.80
Pectine L	5.35	5.08	5.51	4.90	5.08	4.37	4.25
Pectine W	2.14	1.80	1.88	1.68	1.88	1.27	1.23

These negative changes to the habitat of *Paruroctonusconclusus* sp. nov. will likely be further compounded due to climate change in the Mojave Desert. Typical summer daytime high temperatures in Fremont Valley are projected to increase by ca. 6 °C by 2100; furthermore, the frequency of extreme heat days is projected to increase by 8–15 times compared to pre-1990 levels (RWMG 2019). We hypothesize that *P.conclusus* sp. nov. is restricted to this small lakeside area due to the soft soils and increased moisture providing shelter from the daytime heat. Historically, decreases in water levels and increases in temperature have coincided with range reductions and die-offs in desert flora and fauna species associated with playa habitats (Stoffer 2004). This trend is likely to apply to *P.conclusus* sp. nov. as well. Fortunately, the known range of *Paruroctonusconclusus* sp. nov. is entirely on lands managed by the Bureau of Land Management (BLM), meaning that these lands may be eligible for protection. We urge the BLM to consider creating a conservation area for *P.conclusus* sp. nov. and work towards reducing external threats to its habitat.

##### Etymology.

The specific epithet *conclusus* translates to restricted or confined, in reference to the high degree of habitat specialization and severely limited range of *Paruroctonusconclusus* sp. nov.

## ﻿Discussion

Most low-elevation desert *Paruroctonus*, including all California species with the exception of *P.variabilis* and *P.becki*, specialize in habitats of increased moisture such as sand dunes, springs, or alkali-sinks (Gertsch and Soleglad 1966; Haradon 1985; Fet et. al 1998). *Paruroctonussoda* sp. nov. and *Paruroctonusconclusus* sp. nov. both inhabit alkali-sink/playa environments, making them the second and third species in the state to specialize in this habitat. A morphological comparison of all three reveals some important shared characteristics:

1. A reduction in setation: In comparison to other *Paruroctonus*, the number of macrosetae on the pedipalp chela, pedipalp patella, and metasoma is moderately to heavily reduced on alkali-sink species. This is most prominent in *P.bantai* and *P.soda* sp. nov., both of which entirely lack large macrosetae on the manus and have high degrees of macroseta reduction on the pedipalp patella and metasoma. *P.conclusus* sp. nov., also has a moderate degree of macrosetal reduction in these areas (Haradon 1985). Superior macrosetae on the basitarsi and retrosuperior macrosetae on the telotarsi are also slightly reduced. The difference is most prominent when these species are compared to dune-dwelling *Paruroctonus*, which have especially hirsute legs as an adaptation to a psammophilous lifestyle (Haradon 1984a, 1984b).

2. A moderate reduction in pigmentation: In comparison to several other *Paruroctonus*, especially those that occur outside of desert regions such as *P.silvestrii*, *P.boreus*, and *P.maritimus*, these alkali-sink *Paruroctonus* have significant reduction in pigmentation. This is most prominent on the legs, pedipalps, and metasoma, where fuscous markings are entirely absent, but is also the case to a lower degree on the carapace and tergites. The lowest degree of pigment reduction is present in *P.soda* sp. nov. while the highest degree of pigment reduction is present on *P.conclusus* sp. nov. The level of pigment reduction, however, is not as significant as it is in desert dune-dwelling *Paruroctonus* such as *P.xanthus*, *P.luteolus*, or *P.baergi* (Haradon 1984a, 1984b).

3. An enlargement of the chela: the chelae of all three alkali-sink species are very incrassate with heavy scalloping in males, leaving a large proximal gap between the fingers when closed. This is most prominent in *P.soda* sp. nov. and *P.conclusus* sp. nov. While some other species, such as *P.boreus*, *P.arenicola*, and *P.baergi* also possess a similar level of chelal enlargement, especially in males, they are in the minority (Haradon 1983, 1984a, 1984b, 1985).

**Figure 28.**
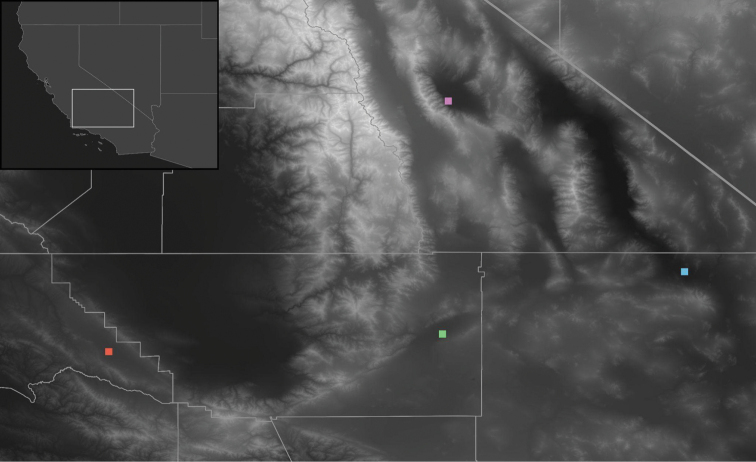
Distribution overview of the 3 Alkali sink *Paruroctonus* species: *P.soda* sp. nov. (red), *P.conclusus* sp. nov. (green), *P.bantaibantai* (purple), and *P.bantaisaratoga* (blue). Enlarged map represents the rectangle on the inset map at the top left, shading represents elevation with dark being low and light being high elevations, and lines represent county borders.

Most estimates place the formation of the Tehachapi mountains and Sierra Nevada separating the San Joaquin Desert from the Mojave Desert in the Eocene (Buwalda 1954; Mix et al. 2016), far before the formation of the Soda Lake endorheic basin in the Pliocene. Furthermore, alkali sinks typically form in endorheic basins, which in most cases require surrounding mountain ranges to prevent outflow to the ocean. It is unlikely that these alkali-sink specialist *Paruroctonus* species would have been able to traverse a mountain range, suggesting they likely speciated as the present day geographies of California emerged (Fig. 28). The *Paruroctonus* with the greatest degree of morphological similarity to these three alkali sink species is *P.boreus*, a species which can also be found in higher-elevation alkali sink areas such as the Deep Springs Valley in California or the Great Salt Lake region in Utah. The three alkali sink *Paruroctonus* may have shared a common ancestor with *P.boreus* that specialized in mountainous habitats. In times of cooler temperatures or higher moisture, such a species’ range could extend into lowland areas, subsequently contracting as temperatures warmed and conditions dried. This range contraction would have resulted isolated populations in alkali sink areas which would eventually diverge into distinct species while still exhibiting either plesiomorphic morphology or convergent evolution from similar ecological constraints. Such a process may be ongoing in populations of *P.boreus* in areas such as Deep Springs Valley.

*P.soda* sp. nov. and *P.bantai* share a much higher degree of morphological similarity than either does with *P.conclusus* sp. nov. This includes very similarly reduced pedipalp and metasomal setation and more similar patterns of fuscousity. However, *P.soda* sp. nov. and *P.bantai* are separated by many more geographic barriers (in the form of mountain ranges) from each other than either is from *P.conclusus* sp. nov. Future phylogenetic work on the group will be important to determine not only the relationships between these three species, but also the relative influence of past geologic events on the speciation of the genus *Paruroctonus*. The existence of these species in remnant alkali sinks mean that their distributions are extremely restricted, and their conservation should be prioritized alongside the preservation of their habitat.

### ﻿Additional material examined

#### Paruroctonusvariabilis

USA • 1 ♀ 1 ♂; California, Kern County, Bitter Creek NWR, Klipstein Cyn Rd; 34.9512, -119.4064; 929 m a.s.l.; 29 May 2021; collector leg Harper Forbes, Prakrit Jain; collected at night using handheld UV light. • 1 ♂; California, Fresno County, along W Whitesbridge Rd near Mendota; 36.7285, -120.2964; 50 m a.s.l.; 15 Jul. 2021; collector leg Harper Forbes, Prakrit Jain; collected at night using handheld UV light. • 3 ♀; California, Fresno County, Silver Creek near Panoche; 36.5717, -120.7024; 239 m a.s.l.; 5 Oct 2019; collector leg Harper Forbes, Prakrit Jain; collected at night using handheld UV light. • 1 ♂; California, Contra Costa county, Empire Mine Road near Antioch; 37.9396, -121.8026; 113 m a.s.l.; 24 Sep 2021; collector leg Prakrit Jain; collected at night using handheld UV light. • 1 ♀; California, Kings county, Jackson avenue near Lemoore; 36.2519, -119.8059; 66 m a.s.l.; 3 Sep 2021; collector leg Prakrit Jain; collected at night using handheld UV light.

#### Paruroctonusbecki

USA • 2 ♂; California, Kern County, southeastern edge of Koehn Lake; 35.3123, -117.8614; 581 m a.s.l.; 3 July 2021; collector leg Prakrit Jain; collected at night using handheld UV light • 2 ♀; California, Los Angeles County, Wilsona Gardens; 34.6804, -117.8278; 798 m a.s.l.; collector leg Harper Forbes, Prakrit Jain; collected at night using handheld UV light.

#### Paruroctonusmarksi

USA • 1 ♂ 1 ♀; California, Los Angeles County, Wilsona Gardens; 34.6804, -117.8278; 798 m a.s.l.; collector leg Harper Forbes, Prakrit Jain; collected at night using handheld UV light.

#### Paruroctonusboreus

USA • 3 ♂ 1 ♀; California, Alpine County, W side Monitor Pass, Hwy 89 × 4 intersection; 38.6605, -119.7264; 1738 m a.s.l ; 15 Sep. 1980; collector leg Stan C. Williams. • 1 ♂ 3 ♀ ; California, Alpine County, W. side along Hwy 89; 18 Jun. 1980; collector leg Stan C. Williams. • 1 ♀; California, Inyo County, White Mountains, Sierra Vista; 37.3563, -118.1868; 2836 m a.s.l.; 12 Jun. 2020; collector leg Harper Forbes, Prakrit Jain; flipped under rocks. • 2 ♀; California, Inyo County, Big Pine Creek Campground; 37.1254, -118.437; 2377 m a.s.l.; 11 Jun. 2020; collector leg Harper Forbes, Prakrit Jain; collected at night using handheld UV light. • 2 ♀; California, Inyo County, Deep Springs Lake; 37.2866, -118.0395; 1501 m a.s.l.; 13–15 Jun. 2021; collector leg Harper Forbes, Prakrit Jain; flipped under debris. • 1 ♂; Nevada, Lander County, Pete’s Summit; 39.1848, -116.7914; 2420 m a.s.l.; 4 July 2021; collector leg Corey Lange; collected at night using handheld UV light.

#### Paruroctonussilvestrii

USA • 1 ♀; California, Colusa County, Bear Creek; 38.9772, -122.3391; 302 m a.s.l.; 15 May 2021; collector leg Harper Forbes, Prakrit Jain; collected at night using handheld UV light. • 1 ♂; California, Orange County, Santa Ana Mountains, Lost Woman Cyn; 33.7525, -117.5513; 767 m a.s.l.; 4 August 2021; collector leg Harper Forbes, Prakrit Jain; collected at night using handheld UV light. • 1 ♀; California, Stanislaus County, Del Puerto Canyon Road; 37.4750, -121.2388; 107 m a.s.l.; 1 March 2020; collector leg Harper Forbes; flipped under rocks. • 1 ♂; California, Alameda County, Patterson Pass Road; 37.6961, -121.5894; 235 m a.s.l.; 26 June 2021; collector leg Harper Forbes, Prakrit Jain; collected at night using handheld UV light. • Mexico • 2 ♂, 2 ♀; Baja California Norte, Puerto Santo Tomas; 8 m a.s.l.; 11 Jul. 1969; collector leg Stan C. Williams, V. F. Lee.

#### Paruroctonusbantai

USA • 1 ♀; California, Inyo County, Saline Valley, west of dunes; 36.7500, -117.8617; 350 m a.s.l.; 14 June 2020; collector leg Harper Forbes, Prakrit Jain; collected at night using handheld UV light.

## Supplementary Material

XML Treatment for Paruroctonus soda

XML Treatment for Paruroctonus conclusus
